# Optimizing DSSCs Performance for Indoor Lighting: Matching Organic Dyes Absorption and Indoor Lamps Emission Profiles to Maximize Efficiency

**DOI:** 10.1002/open.202400464

**Published:** 2025-01-28

**Authors:** Giorgia Salerno, Daniele Franchi, Alessio Dessì, Matteo Bartolini, Norberto Manfredi, Alessandro Abbotto, Ottavia Bettucci

**Affiliations:** ^1^ Department of Materials Science Solar Energy Research Center MIB-SOLAR and INSTM Milano-Bicocca Research Unit University of Milano-Bicocca,Via Cozzi 55 Milano I-20125 Italy; ^2^ Department of Information and Electrical Engineering and Applied Mathematics (DIEM) University of Salerno Invariante 12/B, Via Giovanni Paolo II, 132 Fisciano (SA) I-84084 Italy; ^3^ National Council of Research – Institute of Chemistry of Organometallic Compounds (CNR-ICCOM) Via Madonna del Piano 10 Sesto Fiorentino 50019 Italy

**Keywords:** Solar cells, Indoor photovoltaics, Organic dyes, Matching dye-lamp, Low-cost solar devices

## Abstract

The rapid proliferation of internet‐connected devices has transformed our daily habits prompting a shift towards greater sustainability in renewable energy for indoor applications. Among the various technologies available for obtaining energy in indoor conditions, Dye‐Sensitized Solar Cells (DSSCs) stand out as the most promising due to their ability to efficiently convert ambient light into usable electricity. This study explores how the optimal matching of the UV‐Vis absorption spectra of dyes commonly used in DSSCs with the emission profiles of indoor lamps allows for the enhanced efficiency of DSSC under indoor lighting. By testing four organic dyes with different UV‐Vis absorption spectra (**L1**, **Y123**, **S1**, and **TP1**) under two different common indoor light sources (OSRAM 930 and OSRAM 765 lamp), a significant dye‐lamp correlation was demonstrated. Notably, low‐priced dyes like **S1** and **TP1**, characterized by easier synthetic routes and with an optimal overlap with the dye‐lamp spectrum, exhibited competitive efficiencies, narrowing the performance gap with high‐performing dyes like **Y123**, which require more demanding preparation approaches. The study highlights the critical importance of tailoring dye selection to specific indoor lighting environments, addressing a significant gap and paving the way for more sustainable and cost‐effective energy solutions for indoor applications.

## Graphical abstract

The study highlights the importance of matching dye and light source in optimizing the DSSCs performance under indoor lighting. Improved efficiency is observed when dyes with UV‐Vis spectra match the specific optical characteristics of the light source. The simpler, cost‐effective dye **TP1** demonstrated an excellent cost‐performance balance, suggesting that optimal matching allows for high DSSC efficiency while maintaining affordable synthetic accessibility.

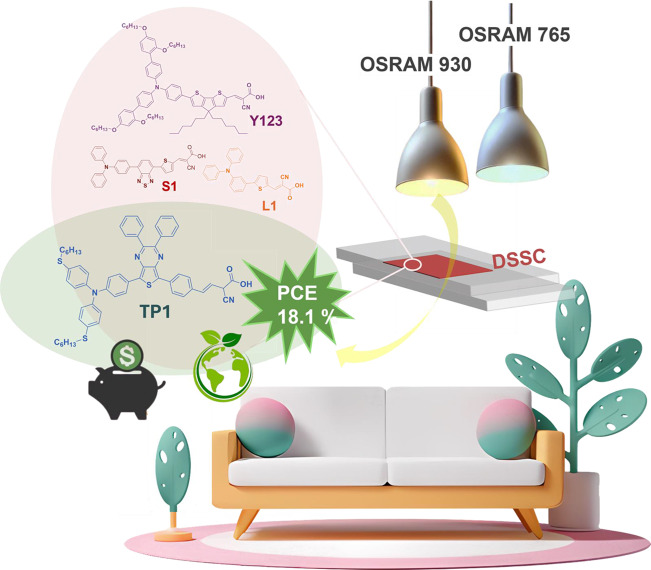

## Introduction

In the last decade, the demand for external energy to power indoor devices dramatically increased with the growing reliance on technology, especially with the rise of the Internet of Things (IoT). We are witnessing a change in our habits due to the rapid increase of a network of internet‐connected physical devices, equipped with autonomous and intelligent sensors, which aim to enhance information exchange in homes, offices, and cities. For this reason, greater emphasis is being placed on achieving complete sustainability in renewable energy sources for indoor applications. Similarly, to what happens for outdoor applications, the integration of organic molecules into sustainable devices (such as photovoltaics,^[1]^ photocatalytic hydrogen production,^[2]^ and electro‐ or photocatalytic ammonia production^[3]^) is increasingly being adopted also to develop indoor devices to make the entire device lifecycle environmentally friendly. Indeed, the use of organic molecules reduces the dependence on critical, rare, expensive, or toxic materials that are often used in traditional energy devices or catalysts. Furthermore, the tunability and cost‐effectiveness of organic molecules make them highly appealing for advancing sustainable energy technologies. Among all the technologies available for obtaining energy in indoor conditions, photovoltaic technology stands out as the most promising due to its ability to efficiently convert ambient light into usable electricity.^[4]^ Intending to combine indoor photovoltaics and organic materials, it is obvious that the most promising devices are Dye‐Sensitized Solar Cells (DSSCs).^[5]^ The use of DSSCs for capturing solar energy indoors is indeed gaining popularity as a sustainable energy solution for everyday applications due to the possibility of being customized^[6]^ by selecting organic dyes designed “ad hoc” in terms of shape, color, and size.^2^, ^7^

**Figure 1:**
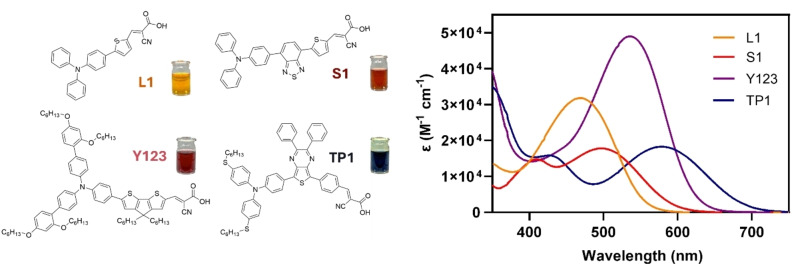
Dyes tested in this work and their UV‐Vis absorption spectra (recorded in CH_2_Cl_2_ solutions).

Moreover, from a sustainability perspective, the use of Deep Eutectic Solvents (DESs) has been recently explored as electrolytes in DSSCs, offering a promising alternative to toxic and flammable Volatile Organic Compounds (VOCs) due to their low volatility, high thermal stability, nonflammability, low cost, and customizable properties.^[8]^ Additionally, unlike conventional silicon‐based solar cells, DSSCs can perform very well in low‐light conditions due to their superior ability to capture diffuse light.^4^, ^9^ Another advantage of DSSCs is their design flexibility, offering transparency and structural adaptability, making them ideal for indoor integration and particularly attractive to power small electronic devices and sensors, contributing to emissions reduction by providing decentralized, renewable energy sources. The dye (photosensitizer) is indeed a key component in a DSSC absorbing light and injecting electrons into the conduction band of the semiconductor, which then transport electrons to the electrode and, from here, to the external circuit, powering devices.^[10]^ The oxidized dye, generated after electron donation to the semiconductor, must efficiently receive electrons from a redox shuttle to minimize the competitive electron back‐transfer process from the semiconductor.^[11]^ Thanks to these multiple capabilities, the proper choice of dyes allows us to obtain highly performing DSSCs. For this reason, many studies are focused on optimizing their structures.^[12]^ For outdoor applications, an extensive strategy to maximize the energy conversion efficiency of DSSCs involves designing dyes that can absorb photons across the entire spectrum of the solar emission, extending from the UV‐visible to the near‐infrared (NIR) region.^[10]^ However, for indoor applications, it is necessary to consider that each environment has different lighting, and each lamp has a characteristic emission spectrum and intensity^[13]^ (i. e., light‐emitting diode‐LED, compact fluorescence‐CF, and Neon lamps, have illuminance values ranging from 200 to 1000 lux while the outdoor solar AM 1.5 G light has an illuminance value of approximately 100,000 lux).^[14]^ Therefore, it is highly strategic that, for indoor applications, the chemical‐physical features of the dyes must be optimized primarily based on the light source used.^[15]^ Nevertheless, to date, no studies on dye‐lamp matching have been conducted, and it is often assumed that dyes performing well in outdoor conditions (AM 1.5G) or with a specific indoor lamp will also provide good efficiencies in common indoor environments, regardless of the specific lamp used in real‐world settings. For this reason, most studies are performed using only one light source (typically OSRAM warm white 930) reaching notable results but often using dyes with complex structures, which require not cost‐effective multistep syntheses. Conversely, to optimize the performance of DSSCs for indoor applications, it is necessary to establish a relationship between the UV‐Vis emission spectra of indoor light sources and the absorption profiles of the organic dyes to predict the best overlap between the dye absorption and lamp emission spectra.^[16]^ This correlation would help to identify which dye is the most suitable for a certain light source, regardless of its behavior in outdoor conditions. Based on our hypothesis, the ideal approach would be to use organic dyes that can be obtained by simple synthetic processes, but also exhibit a good match between their UV‐Vis absorption and the emission profile of the selected light source, to save time and synthesis costs without compromising the device's performance making the entire process more sustainable and effective. To ascertain this hypothesis, this study tested different organic dyes, **L1**,^[6e]^ **Y123**,^[10]^ and **S1**,^[17]^ previously reported in the literature, and one dye, **TP1**, that some of us have recently designed for DSSC under outdoor standard conditions (1 sun).^[18]^ The dyes were chosen as they exhibit representatively different absorption profiles (Figure 1) and, accordingly, different matching with two light sources. The spectra in Figure 1 are plotted as a function of the molar extinction coefficient (ϵ) to highlight not only the band profile but also the different molar absorptivities. We have selected, as common representative indoor warm and cold lamps, OSRAM 930 (warm light T8 fluorescent lamp, OSRAM L 18 W/930) and OSRAM 765 (cold light T5 fluorescent lamp – OSRAM L 8 W/765), respectively. Both sources are typically used for indoor lighting in supermarkets, offices, hospitals, etc. This study has been focused to two representative light sources commonly found in indoor settings and frequently reported in the literature. This decision allowed us to focus on establishing a foundational understanding of the performance of the dyes under controlled conditions. Moreover, the significantly different emission profiles of the two lamps allowed to capture a broad range of potential indoor lighting conditions. The different behavior shown by the dyes in both lighting conditions allows a consistent correlation between the dye absorption spectrum and the light emission profiles demonstrating that a good dye‐lamp match can optimize the performances of DSSCs in indoor conditions independently from the chemical structure of the dyes and their performances in outdoor conditions filling a significant knowledge gap.

## Results and Discussion

### Qualitative and Quantitative Dye‐Lamp Matching

**Figure 2:**
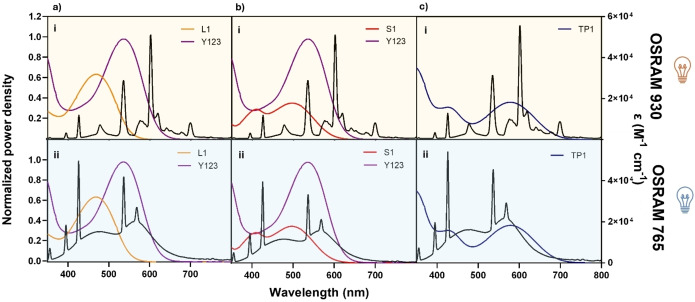
Overlap between UV‐Vis absorption spectra of dyes (CH_2_Cl_2_ solutions) and normalized (emission peak) emission profiles of OSRAM 930 and OSRAM 765 lamps.

The four dyes chosen for this study were carefully selected to demonstrate the correlation between the emission profiles of the lamps and the dye's UV‐Vis absorption spectra. First, all the dyes have been synthesized following the synthetic pathways reported in the literature^6^, ^10^, ^17^, ^18^ and optically characterized to obtain the UV‐Vis spectra profiles both in solution and as a solid film on TiO_2_ (Figures S1 and S2 and Table S1). A qualitative estimation of the dye‐lamp matching was carried out by comparing the dye absorption spectra (ϵ vs. λ) with the emission profiles of the lamps in the 350–800 nm wavelength region, measured using a Hamamatsu C10082CAH spectrophotometer and a power meter (Figure 2). Based on a preliminary evaluation, Figure 2 shows that all of the dyes show a better overlap with the OSRAM 765 lamp emission. Furthermore, the emission peak of the OSRAM 930 lamp falls, in all cases except for **TP1**, within a region where the dye either does not absorb or has very low absorption (lower‐energy absorption profile).

More in details, when **L1**, with a very simple structure and easy synthesis, and **Y123**, with a more complex structure and more demanding synthetic approach, have been compared (Figure 2a), they show significant differences in light absorption resulting in a crucial disparity in the overlap with the emission profiles of the light sources. Indeed, dye **Y123** has a more panchromatic and red‐shifted absorption, covering a significant portion of the spectrum of both lamps, fitting better with the emission peaks of the OSRAM 765 (Figure 2a–ii). This different behavior in terms of lamp matching is even more evident for dye **L1**, which covers a significant portion of the emission spectrum of the OSRAM 765 lamp, including the most intense emission peak (Figure 2a–ii), and no important emission peaks of the OSRAM 930 lamp (Figure 2a–i). This strong overlap difference between lamp emission and dye absorption spectra allows us to obtain valuable information to validate the hypothesis of dye‐lamp matching. The UV‐Vis absorption spectrum of a third dye with a simple chemical structure, **S1**, which has never been tested before for indoor applications, was also examined (Figure 2b). Dye **S1** exhibits an absorption profile similar to that of **Y123** and we expect similar behavior when exposed to the indoor lighting. Therefore, it is expected to perform better with the OSRAM 765 lamp where a better overlap is present (Figure 2b–ii). Thanks to the similarity in the absorption profile spectra of these two dyes it is possible to determine whether a simple dye structure as **S1** can perform in indoor lighting similarly to a more complex and expensive (in terms of synthetic route) dye structure, such as **Y123**. Lastly, a fourth dye, **TP1**, recently introduced by some of us and never tested before under indoor conditions, was chosen because of its notable red‐shift in its absorption spectrum. This fascinating feature provides a better overall matching with both lamps compared to all the previously discussed dyes (Figure 2c). Moreover, the qualitative analysis depicted in Figure 2c suggests that **TP1** is the only dye among those examined in this work that shows a good overlap with the OSRAM 930 lamp (Figure 2c–i). These results align with the scope of this work, focusing on optimal dye‐lamp profiles. Indeed, **TP1** is the only dye, amongst the four investigated in this work, with a significant absorption (close to the absorption peak) at the wavelength corresponding to the maximum emission peak of the OSRAM 930 lamp. By testing the efficiency of photovoltaic devices sensitized by these dyes, it is possible to validate whether a simple dye structure with a broad UV‐Vis absorption performs better than a dye with a lower dye‐lamp matching (**S1** and **L1**) and whether its performance is comparable to that of more complex dyes such as **Y123**, in particular by exploiting its better overall matching with both lamps. A comparison of UV‐Vis absorption profiles of the dyes adsorbed onto TiO_2_ films and emission spectra of the two light sources is also shown in Figure S4.

So far, the evaluation of the matching between the dye's absorption spectrum and the lamp's emission spectrum has been primarily qualitative, which can be subject to personal interpretations and therefore not universally valid. To enable a quantitative correlation with photovoltaic data, it is necessary to develop a new quantitative assessment that parametrizes the best matching between the optical properties of the dye and the lamp. Therefore, we have developed a quantitative analysis of the portion of the emission profile of the lamps covered by the absorption spectra of each dye.

First, we have calculated the oscillator strength “*f*” (350–800 nm region) for each dye (eq. 1

(1) $$f=\left(\frac{4m_{e}c\epsilon_{0}}{N_{A}e^{2}}ln10\right)\times \;A\;$$

where A is the integrated absorption coefficient and the term in parenthesis is equal to $1.44\times 10^{-19}$. Once this value is obtained, we define a *weighted* oscillator strength (*f’*) that accounts for the matching with the lamp's emission spectrum. The value of *f’* is calculated from the *weighted* integrated absorption coefficient A', derived from the molar absorptivity spectrum of the dye where the extinction coefficient for each dye is *weighted* (new value ϵ’) based on the lamp's emission spectrum. Specifically, at the peak of the lamp's emission spectrum, the molar absorptivity of the dye remains unchanged (ϵ’=ϵ), while for other wavelengths it is scaled by a factor corresponding to the ratio of the lamp's emission intensity at that wavelength to its peak emission intensity. This approach yields new values of *weighted* molar absorptivity ϵ’ recalibrated according to the lamp's emission spectrum. Table S2 presents the detailed calculations to obtain the ϵ’ values (illustrated with an example for dye **L1** and the OSRAM 765 lamp). The ϵ’ vs. λ spectra are shown in Figure S5. In this way, *f’* values weighted to the lamp's emission spectrum (*f’*_765_ and *f’*_930_) were obtained. Finally, the ratio between *f* weighted (*f’*) and *f* real has been used as a quantitative matching parameter. The real *f*, the weighted *f’*, and the *f’*/*f* ratio values for each dye are listed in Table 1. Table 1 also presents the ratio of the *f*’ values for the two lamps (*f*′_765_/*f*′_930_), indicating the lamp with the best match and quantifying the extent of this differing behavior.

**Table 1:** Oscillator strength *f* (real) and *f’* (weighted, based on the lamp's emission spectrum) values for the investigated dyes (350–800 nm region).

Dye	f	f′_930_	f′_765_	f’_930_/f	f′_765_/f	f’_765_/f’_930_
**L1**	0.91	0.0073	0.20	8.0×10^−3^	2.2×10^−1^	27
**S1**	0.62	0.0087	0.14	14×10^−3^	2.3×10^−1^	16
**Y123**	1.40	0.038	0.34	27×10^−3^	2.5×10^−1^	9
**TP1**	0.96	0.035	0.18	36×10^−3^	1.9×10^−1^	5

Table 1 quantitatively confirms the previous qualitative analysis, demonstrating that the OSRAM 765 lamp consistently shows a higher *f*’ value for all dyes, indicating a better match. However, when comparing the *f*′_765_/*f*′_930_ ratios, this value is considerably higher for **L1**, **S1**, and, to a lesser extent, **Y123**, indicating a significantly better matching with the OSRAM 765 lamp optical characteristics. In contrast, the ratio is more modest for dye **TP1**, suggesting that **TP1** overlaps well with both lamps, consistent with the qualitative findings. Accordingly, in the experimental DSSC tests, we expect to record higher short‐circuit currents *J*_sc_ and power conversion efficiencies (PCEs) with the OSRAM 765 lamp, especially for the first three dyes, whereas a somewhat different behavior is expected for **TP1**. It is important to note that this approach, though quantitative, is not meant to provide a linear relationship with experimental photovoltaic parameters, such as PCE or even the photocurrents *J*_sc_. Instead, it should be treated as a trend to numerically assess where the best matching occurs between the dyes and the lamps, as well as a tool for comparing the best matching of different dyes or different lamps.

### Indoor Photovoltaic Performances

DSSCs were fabricated using a 10‐μm thick TiO_2_ layer (Figure S6) deposited on a glass coated with FTO. Dye sensitization was obtained by immersing the semiconductor layer in the dye solution overnight. A poly(3,4‐ethylenedioxythiophene (PEDOT) counter electrode has been manufactured *via* electro‐polymerization and assembled with the dye‐sensitized TiO_2_ electrode into a sealed sandwich‐type cell using standard thickness commonly reported in recent works on high performing indoor DSSC.^[6e]^ The cell was then filled with a solution of a redox pair, Cu^I^(tmby)_2_TFSI and Cu^II^(tmby)_2_(TFSI)_2_ (tmby=4,4′,6,6′‐tetramethyl‐2,2′‐bipyridine; TFSI=bis(trifluoromethanesulfonyl)imide).^[19]^ The selection of the copper‐based redox couple was motivated by the superior performances reported in the recent literature in highly efficient indoor DSSC studies, which outperform both iodine‐ and cobalt‐based electrolytes thanks to the lower reorganization energy and minimized overpotential losses.^19^, ^20^ Cu^I^/Cu^II^ complexes have been synthesized following the literature procedure (see Supporting Information),^[20]^ fully characterized through cyclic voltammetry and UV‐Vis spectroscopy (Figures S7 and S8 and Table S3),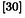 and freshly synthesized before each use. First, all dyes were tested under conventional AM 1.5 G sun‐simulated light. The two dyes **S1** and **TP1** have never been tested in DSSCs with copper‐based electrolytes. All dyes showed PCE values in line with the literature data (between 3 and 5 %) (Figure S9 and Table S4). No significative differences have been observed for dyes **L1**, **S1**, and **TP1** which showed efficiencies of 4.0, 3.8 and 3.6 %, respectively.^6^, ^10^, ^17^, ^18^ A slightly higher efficiency of 4.6 % was obtained for **Y123**. All data on *J/V* characteristics obtained under AM 1.5 G sun‐simulated light are summarized in Table S4.

**Figure 3:**
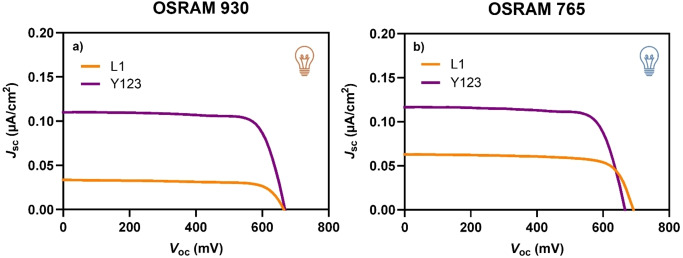
*J*/*V* curves of DSSCs sensitized by dyes **L1** and **Y123** under OSRAM 930 (a) and OSRAM 765 (b) illumination (1000 lux).

The selection of dyes, all operating within the same efficiency range under standard conditions, was made to better analyse the differences observed when switching to indoor lighting and varying lamps. The corresponding dye‐sensitized devices were then tested under the two light sources (OSRAM 930 and OSRAM 765). First, devices based on dyes **L1** and **Y123** were compared. The *J/V* curves of the devices sensitized with the two dyes showed significantly different behaviour under the OSRAM 930 lamp (Figure 3a), with a maximum PCE value of 18.6 % for **Y123**‐sensitized DSSC and 5.5 % for **L1**‐sensitized DSSC. The better efficiency for the former cell originates from a higher *J*_sc,_ which, in fact, is reflected in the improved *f* value and better overlap observed in the qualitative analysis (Figure 2a–i and Table 1). Conversely, when the OSRAM 765 lamp was used, where both dyes show better lamp matching (Figure 2a–ii), it is observed that **Y123** slightly improves its efficiency to 19.1 %, while **L1** notably doubles its efficiency, reaching a remarkable PCE of 10.9 % (Figure 3b). Remarkably, Table 1 shows that the *f’*_765_/*f’*_950_ for **L1** is significantly higher, indicating that switching from the OSRAM 930 to the OSRAM 765 lamp provides the greatest improvement for this dye. This observation is elegantly confirmed by the photovoltaic measurements, which show a two‐fold enhancement in PCE. The result provides a first strong evidence of the crucial role of dye‐lamp matching in DSSCs for indoor applications. All data on *J/V* characteristics are summarized in Table 2.

**Table 2:** *J*/*V* characteristics of DSSCs sensitized by dyes **Y123** and **L1** under OSRAM 930 and OSRAM 765 illumination (1000 lux).^[a]^

Dye	OSRAM 930					OSRAM 765				
	V_oc_ (mV)	J_sc_ (μA/cm^2^)	FF	P_max_ (μW/cm^2^)	PCE (%)	V_oc_ (mV)	J_sc_ (μA/cm^2^)	FF	P_max_ (μW/cm^2^)	PCE (%)
**L1**	664 (658±5)	33.5 (32.0±1.8)	0.75 (0.72±0.03)	16.7 (15.2±1.4)	5.5 (5.0±0.5)	645 (672±24)	68.6 (64.0±4.2)	0.76 (0.77±0.04)	33.6 (33.0±0.7)	10.9 (10.7±0.2)
**Y123**	668 (669±22)	110.3 (102.1±7.9)	0.77 (0.77±0.01)	56.7 (52.6±3.6)	18.6 (17.3±1.2)	666 (667±21)	116.7 (108.8±7.5)	0.76 (0.76+0.01)	59.1 (55.4±3.2)	19.1 (17.9±1.0)

^[a]^ Average values over 3 devices in parentheses.

**Figure 4:**
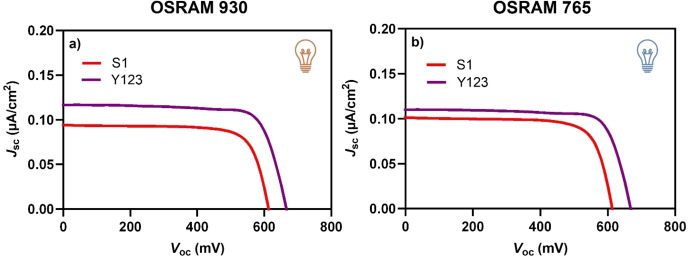
*J*/*V* curves of DSSCs sensitized by dyes **S1** and **Y123** under OSRAM 930 (a) and OSRAM 765 (b) illumination. (1000 lux).

In the case of the dye **S1**, the UV‐Vis absorption spectrum as a TiO_2_ film is similar to that of dye **Y123** (Figure S1). However, the chemical structures are very different. The structure of dye **S1** is much simpler and requires fewer synthetic and purification steps compared to the more sophisticated **Y123**. In agreement with the more similar absorption profiles, measured photocurrents *J*_sc_ were comparable for devices based on these two dyes (Figure 4). It can be observed that DSSCs sensitized by **S1** achieved a PCE of 14.2 and 15.0 % with the OSRAM 930 and 765 lamps respectively (Table 3), which are very similar values, in contrast to the significant different values recorded for dye **L1**. These results align well with the dye‐lamp matching discussion, as the UV‐Vis spectra of dye **S1** show a smaller difference in overlap (*f’*_765_/*f’*_930_) between the two lamps compared to dye **L1**, which exhibits a more pronounced disparity (Figures 2a–b and Table 1). However, despite the similar UV‐Vis absorption profiles of **S1** and **Y123**, DSSCs sensitized by the latter dye afforded higher PCEs (18.6 and 19.1 % with the OSRAM 930 and 765 lamps, respectively; Table 3). The superior PCE of **Y123**‐sensitized cells stems from a higher photocurrent, due to the increased absorption intensity across the entire spectrum (f) (Figure 1 and Table 1). Additionally, **Y123**‐based devices exhibit higher open‐circuit voltages *V*_oc_. Under 1 sun illumination, the PCE of **S1** based cells is more similar to that of **L1** (Figure S9; Table S4). However, when tested under indoor conditions, the same device narrows the gap with **Y123**‐based DSSCs, thanks to the proper dye‐lamp matching of **S1**, thus making this less complex dye competitive in terms of efficiency. All data on *J/V* characteristics are summarized in Table 3.

**Table 3:** *J*/*V* characteristics of DSSCs sensitized by dyes **S1** and **Y123** under OSRAM 930 and OSRAM 765 illumination (1000 lux).^[a]^

Dye	OSRAM 930					OSRAM 765				
	V_oc_ (mV)	J_sc_ (μA/cm^2^)	FF	P_max_ (μW/cm^2^)	PCE (%)	V_oc_ (mV)	J_sc_ (μA/cm^2^)	FF	P_max_ (μW/cm^2^)	PCE (%)
**S1**	613 (625±14)	94.1 (84.9±9.3)	0.75 (0.76±0.02)	43.3 (40.1±2.9)	14.2 (13.2±0.9)	613 (629±19)	101.3 (94.1±7.3)	0.75 (0.75±0.02)	46.6 (44.3±2.0)	15.0 (14.3±0.6)
**Y123**	668 (669±22)	110.3 (102.1±7.9)	0.77 (0.77±0.01)	56.7 (52.6±3.6)	18.6 (17.3±1.2)	666 (667±21)	116.7 (108.8±7.4)	0.76 (0.76±0.01)	59.1 (55.4±3.2)	19.1 (17.9±1.0)

^[a]^ Average values over 3 devices in parentheses.

Finally, Table 4 collects the photovoltaic data of DSSCs based on **TP1** under indoor conditions. It should be emphasized that **TP1** is the only dye among those investigated that showed an efficient overlap with both lamps (Figure 2c), as confirmed by its low *f’*_765_/*f’*_950_ ratio. This low ratio can be interpreted as indicating minimal difference in optical matching between the dye and the two lamps. This may explain why, although slightly, a higher PCE is observed under OSRAM 930 illumination. In fact, **TP1** is the only dye whose absorption profile overlaps with both of the most intense emission peaks of the OSRAM 930 lamp.

**Table 4:** *J*/*V* characteristics of DSSCs sensitized by dye **TP1** under OSRAM 930 and OSRAM 765 illumination (1000 lux).^[a]^

Dye	OSRAM 930					OSRAM 765				
	*V*_oc_ (mV)	*J*_sc_ (μA/cm^2^)	FF	P_max_ (μW/cm^2^)	PCE (%)	*V*_oc_ (mV)	*J*_sc_ (μA/cm^2^)	FF	P_max_ (μW/cm^2^)	PCE (%)
**TP1**	673 (678±5)	107.9 (98.3±9.0)	0.76 (0.77±0.01)	55.2 (55.1±4.1)	18.1 (16.8±1.3)	667 (670±3)	97.3 (89.1±7.6)	0.76 (0.77±0.01)	49.3 (45.7±3.4)	15.9 (14.8±1.1)

^[a]^ Average values over 3 devices in parentheses.

Although **TP1** has a simpler structure (i. e., requiring fewer synthetic steps and thus cheaper to produce; *vide infra*) than **Y123**, it is not surprising that a good match between its absorption spectrum and the emission spectrum of the lamp yields a very high efficiency (PCE=18.1 %), comparable to that of **Y123**‐sensitized devices. In particular, the short‐circuit current, which directly depends on the optical properties, is nearly identical for the two dyes under the OSRAM 930 lamp. These results, in conjunction with previously data, emphasize the critical importance of optimizing the match between the UV‐Vis absorption spectra of the dye and the emission profile of the lamp for maximizing DSSCs performances under indoor lighting.

**Figure 5:**
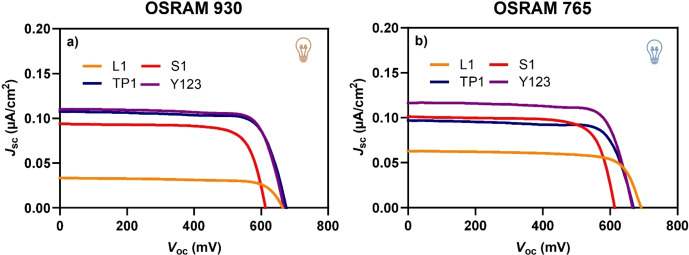
*J*/*V* curves of DSSCs sensitized by all dyes under OSRAM 930 (a) and OSRAM 765 (b) illumination (1000 lux).

Figure 5 shows the *J*/*V* curves and Table 5 summarizes the PCE values for DSSCs sensitized by the four dyes under the two OSRAM lamps for overall comparison. Additionally, Table 5 includes the synthetic accessibility and the final cost of each dye, estimated following the procedure proposed by Osedach et al.^[21]^ and later applied by Nazeeruddin et al.^[22]^ This procedure estimates the synthetic accessibility of a molecule considering the number of synthetic steps and the commercial cost of the starting materials (simple molecules) that are currently available from bulk chemical suppliers (in our case, Merck Sigma‐Aldrich). All the details about the evaluation of the synthetic accessibility and cost for each dye are provided in the Supporting Information (Schemes S1–S4). When the dye itself was commercially available, the commercial price was also reported for clarity. A more detailed quantitative analysis is beyond the scope of this work, as many parameters such as the volume of solvents used in liquid‐liquid extractions and chromatographic processes were not optimized, given that the dyes were not specifically designed for this purpose.

**Table 5:** Summary of PCE values under OSRAM lamps^[a]^ in combination with estimation of synthetic accessibility.

Dye	PCE using OSRAM 930 (%)	PCE using OSRAM 765 (%)	Synthetic steps (literature procedure)^[b]^	Cost (literature procedures)^[b]^		Cost (commercial dyes)^[c]^		Relative molar cost^[d]^
				(EUR/g)	(EUR/mmol)	(EUR/g)	(EUR/mmol)	
**L1**	5.5	10.9	2	42	18	3900	1646	1
**S1**	14.2	15.0	3	511	285	–	–	16
**Y123**	18.6	19.1	12	951	1177	5820	7205	65
**TP1**	18.1	15.9	6	200	187	–	–	10

^[a]^ 1000 lux. ^[b]^ Number of synthetic steps and cost estimated from representative synthesis procedures reported in the literature (see Supporting Information).^[c]^ Dyenamo (https://dyenamo.se) (**L1** Dyenamo code: DN−F02; **Y123** Dyenamo code: DN−F05Y).^[d]^ With respect to **L1**.

Table 5 compares the PCE values of dyes with very different production costs, estimated in different ways. Specifically, it shows that **TP1**‐sensitized DSSC achieves a maximum PCE (18.1 %) very similar to that reached by the corresponding **Y123** device (19.1 %), despite requiring half as many synthetic steps and costing roughly one‐sixth as much. From this perspective, a dye typically considered secondary for real‐world photovoltaic applications due to its lower PCE, like **L1**, still achieves a respectable efficiency (∼11 %) but has an access cost 65 times lower (4 times lower when starting directly from commercial dyes), which naturally suggests that DSSCs based on this dye could still be very attractive from an application standpoint. This analysis clearly demonstrates that a suitable dye‐lamp match can lead to a significant enhancement of DSSC performance in indoor applications, achieving, with low‐cost dyes that have simple synthetic pathways (such as **TP1**), efficiencies comparable to those of very expensive dyes like **Y123**.

## Conclusions

This study successfully elucidates the significance of dye‐lamp matching in optimizing the performance of DSSCs under indoor lighting conditions. The qualitative and quantitative analyses of four selected sensitizers **L1**, **Y123**, **S1**, and **TP1** demonstrate that the efficiency of DSSCs is heavily influenced by the alignment between the dye's UV‐Vis absorption spectra and the emission profiles of the light sources. Devices sensitized by **Y123** exhibited superior performance under both OSRAM 765 and OSRAM 930 lamp due to the superior optical properties (extended π‐conjugated framework) as well as good dye‐lamp matching. The simplest dye, **L1**, with very limited π‐conjugated framework, yielded DSSCs with modest efficiency under the OSRAM 930 lamp. However, through effective dye‐lamp matching, we successfully doubled its efficiency using OSRAM 765, achieving respectable performance levels. The introduction of dye **S1**, which has a UV‐Vis absorption profile similar to that of **Y123** but a simpler structure, demonstrated that the corresponding solar cells achieved competitive efficiencies, confirming that effective absorption can offset structural complexity in dye design. To further support this evidence, a fourth dye, **TP1**, developed by some of us, has been investigated in the same conditions revealing a substantial performance improvement over dyes **L1** and **S1**, while significantly reducing the efficiency gap with dye **Y123**. Such results illustrate that simpler chemical structures can yield remarkable efficiencies when tailored to specific lighting conditions.

**Figure 6:**
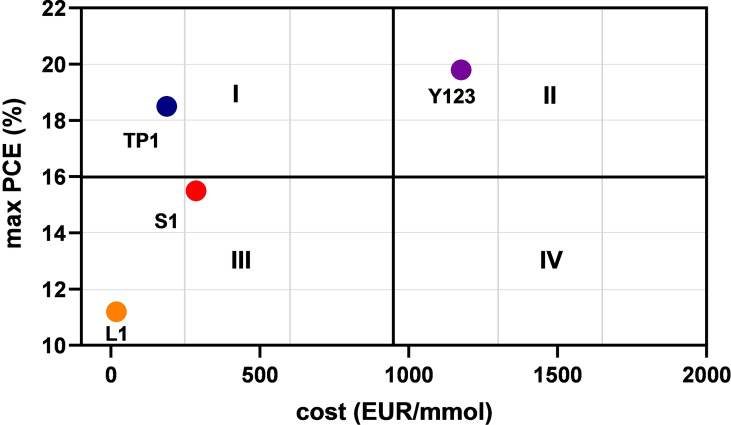
Cost‐PCE analysis of DSSCs based on the four dyes investigated in this study. Quadrant I represents the optimal cost‐PCE balance, while quadrants II and III are considered less efficient compromises. Quadrant IV (higher costs and lower PCE) is notably empty, underscoring the efficiency of the selected dyes.

The search for dyes with maximal PCE should always be accompanied by a thorough cost analysis. In terms of real‐world applications, the device with the highest PCE may not always be optimal if it relies on a complex dye that requires a multistep synthesis, leading to high final costs. It would be preferable to use a DSSC based on a dye that, while offering a similar PCE, is considerably more cost‐effective. In our case, despite using a simpler dye (**TP1**), through optimization of dye‐lamp matching, we achieved an optimal cost‐PCE balance. Figure 6 (cost vs. PCE) illustrates this concept for the dyes studied in this work. Excluding quadrant IV (higher costs and lower PCE, based on our ranges), which remains empty in our study, the best cost‐PCE combination (**TP1** based devices) falls into quadrant I (low costs and high PCE), while quadrants II and III may be considered less efficient compromises. In this context, even a well‐known and straightforward dye, **L1**, with high synthetic accessibility, appears promising. Even though it offers a much lower PCE compared to **Y123**, it remains attractive due to its effective dye‐lamp matching with specific light bulbs. In addition, a new quantitative parameter to assess the alignment between dye absorption and lamp emission has been introduced, allowing for a clear correlation with observed power conversion efficiencies (PCEs). Our results show that DSSCs bearing simpler and more affordable dyes such as **TP1** (up to six times less expensive) can achieve similar performance to those with more complex dyes when paired with suitable indoor light sources. Remarkably, even very low‐cost and basic dyes, like **L1** (first reported in the literature nearly 20 years ago and costing several less than the leading sensitizer, see Table 5), demonstrate respectable efficiencies in comparison to the highest‐performing sensitizers. However, a number of important challenges need to be addressed for commercial‐scale DSSCs including large variability in real‐world indoor lighting, where light sources may largely vary in type, intensity and spectral distribution, even over time. This requires developing a new generation of dyes that can maintain high efficiency across a wide range of conditions, not just under specific, optimized lighting sources. A further typical major challenge is the long‐term stability of both the dye and the whole device under diverse environmental conditions, in order to fully meet commercial general viability.

In conclusion, this study underlines two relevant concepts, dye‐lamp matching and cost‐PCE compromise, suggesting new strategies for the development of high‐efficiency, low‐cost DSSCs suitable for indoor real‐world applications, thus making the entire lifecycle of the device more sustainable in terms of both cost and environmental impact (quantities of input materials for synthesis, amount of waste for unit of produced product, workup and purification costs). Indeed, the dye‐lamp matching could allow for a more economical and environmentally advantageous application of DSSCs in indoor environments. Future steps in this work will explore the possibilities of co‐sensitization between different dyes (with varying structural complexity and optimized cost‐efficiency trade‐offs) to cover a larger portion of the emission spectra of both representative lamps and achieve nearly total overlap, and the use of eco‐friendly solvents (DESs as replacements for VOCs), leading to a notable enhancement of DSSC efficiencies while ensuring low‐cost accessibility and high sustainability.

## Experimental Section

### Reagents and Materials

All reagents were obtained from commercial suppliers at the highest purity grade and used without further purification. Anhydrous solvents were purchased from Sigma‐Aldrich and used without further purification. Extracts were dried with Na_2_SO_4_ and filtered before removal of the solvent by evaporation. Dyes **L1**, **S1**, **Y123** and **TP1** were synthesized according to the literature.^6^, ^10^, ^17^, ^18^ FTO‐coated glass plates, GreatCell Solar 30 NR−D (transparent anatase 30‐nm nanoparticles), and Solaronix Ti‐Nanoxide R/SP (diffusing >100‐nm titania particles for reflective layer) titania pastes have been purchased from commercial suppliers. UV‐O_3_ treatment was performed using Novascan PSD Pro Series‐Digital UV Ozone System. The thickness of the layers was measured by means of a VEECO Dektak 8 Stylus Profiler.

### Solar Cell Fabrication Procedure

DSSCs have been prepared by adapting a procedure reported in the literature.^[23]^ FTO glass plates were cleaned in a detergent solution for 15 min using an ultrasonic bath, rinsed with pure water and cleaned again for 15 min in an ultrasonic bath with EtOH. After treatment in a UV‐O_3_ system for 18 min, a dense TiO_2_ layer was deposited via spray pyrolysis at 450 °C from a 0.02 M titanium tetraisopropoxide and 2 M acetylacetone solution in isopropanol. Subsequently, a layer of 0.20 cm^2^ was screen‐printed using transparent TiO_2_paste (GreatCell Solar 30 NR−D). The coated films were thermally treated at 125 °C for 5 min. Then another layer of TiO_2_paste (Solaronix Ti‐Nanoxide R/SP) was screen‐printed and dried at 125 °C for 5 min. The coated films were thermally treated at 125 °C for 5 min, 325 °C for 10 min, 450 °C for 15 min, and 500 °C for 15 min. The heating ramp rate was 5–10 °C min^−1^. The sintered layer was treated again with 40 mM aqueous TiCl_4_ (70 °C for 30 min), rinsed with EtOH and heated at 500 °C for 30 min. After cooling down to 80 °C, the TiO_2_ coated plate was immersed in the dye solution (**L1**: 0.5 M in CH_3_CN; **Y123**: 0.1 M in *tert*‐butanol:CH_3_CN 1 : 1; **S1**: 0.3 M in THF; **TP1**: 0.1 M *tert*‐butanol:CH_3_CN 1 : 1) for 20 h at room temperature in the dark. PEDOT counter electrodes were manufactured via electro‐polymerization of 3,4‐ethylenedioxythiophene from 0.01 mM aqueous solution with 0.1 M sodium dodecyl sulphate, as reported in the literature.^[24]^ The redox electrolyte solutions were prepared with 0.2 M Cu^I^(tmby)_2_TFSI and 0.04 M Cu^II^(tmby)_2_(TFSI)_2_, 0.1 M LiTFSI and 0.6 M 4‐*tert*‐butylpyridine in CH_3_CN. The dye‐adsorbed TiO_2_ electrodes and the counter electrode were assembled into a sealed sandwich‐type cell by heating with hot‐melt ionomer‐class resin (Surlyn 30 μm thickness) as a spacer between the electrodes. The electrolyte solution was vacuum injected through a hole in the counter electrode which was the sealed with a sheet of Surlyn and a cover glass.

### Solar Cell Measurements at 1 Sun (AM 1.5G)

Photovoltaic measurements of DSSCs were carried out under a 550 W xenon light source (ABET Technologies Sun 2000 class ABA Solar Simulator). The power of the simulated light was calibrated to AM 1.5 G (100 mW cm^−2^) using a reference Si cell photodiode equipped with an IR‐cutoff filter (KG‐5, Schott) to reduce the mismatch in the 350–750 nm region between the simulated light and the AM 1.5 G spectrum.

### Solar Cell Measurements Under Indoor Lighting

Indoor light *J*/*V* curves were obtained using two different light sources: a cold white T5 fluorescent lamp (OSRAM L 8 W/765, abbreviated as OSRAM 765) and a warm white T8 fluorescent tube (OSRAM L 18 W/930, abbreviated as OSRAM 930). In both cases, the light source was placed at a distance to illuminate the surface of interest with an illuminance equal to 1000±50 lux (power density: OSRAM 765, 310 μW/cm^2^; OSRAM 930, 304 μW/cm^2^). Illuminance was measured by using a lux meter (HoldPeak HP‐881 E, accuracy ±4 %) for a fast check during DSSC testing. However, to achieve a more reliable measurement of parameters, PCE was correctly determined by illuminance levels from the irradiance spectra. The emission spectrum of the light source in the wavelength range from 300 to 1000 nm was measured using a Hamamatsu C10082CAH spectrophotometer and a power meter (Thorlabs PM100USB power and energy meter) equipped with a photodiode just calibrated for the purpose (Si‐photodiode S120VC, recalibrated 03/23 by ReRa Solutions). Figure S3 shows the spectral distribution of the irradiance (power per unit illuminated area at the distance of interest). The entire active photovoltaic area of the devices was used during indoor characterization to mimic diffuse light conditions. For each combination of dye/electrolyte, multiple cells have been prepared and tested for average values of 3 independent cells. Listed values included standard errors. Indoor measurements were conducted using two different home configurations (Figure S10). Specifically, the OSRAM lamp (OSRAM 765 or OSRAM 930) was placed inside appropriately sized boxes. The boxes were coated with an opaque black material, and to ensure there were no reflections of light on the photovoltaic device during the measurement, reflectance measurements were taken. These measurements showed that the chosen material does not reflect in the region where the lamp emits (Figure S11). Once the optimal point within the box for making the measurements was identified, as described in the previous paragraph, *J*/*V* curves of the cells were recorded.

### Evaluation of Synthetic Accessibility and Cost

Synthetic accessibility and cost of each dye were estimated following the procedure proposed by Osedach et al.,^[21]^ and subsequently applied by Nazeeruddin et al.^[22]^ Accordingly, we used the number of synthetic steps (that is, the number of explicit isolations of intermediate and products required during the synthetic procedure) as a main parameter for determining synthetic accessibility. To estimate the total production cost of the dye, we have considered the beginning of the process as the point where the starting materials are simple molecules currently available from bulk chemical suppliers. In this work Merck Sigma‐Aldrich has been selected as the main bulk chemical supplier. For each dye we have used a representative synthetic procedure reported in the literature as a reference synthesis (see Supporting Information). For each synthetic step, we identified the quantities of reagents, reactants, and the reaction yields. In this way, the quantities of each reagent needed to produce a specific amount of product were determined. For each step, the prices of reagent were multiplied by the required quantities to determine the material costs. When products with different quantities are available from the selected supplier, the closest larger quantity was chosen compared to that specified in the literature procedure (e. g., if 3.5 g of a reagent was used, we considered the price of the 5 g product, even if a 25 g option is also available). Finally, in order to compare total costs of the different dyes, the calculated values were normalized to 1 g and 1 mmol of each final product. For simplicity, and since it does not significantly alter the conclusions of the analysis, we excluded workup/purification and waste treatment costs (following one of the scenarios described by Osedach et al.).^[21]^ Similarly, costs associated with common, inexpensive laboratory reagents and solvents (inorganic and organic acids and bases like K₂CO₃ or common amines, solvents) were omitted from the total cost calculation.

## Supporting Information Summary

The authors have cited additional references within the Supporting Information.^[25]^

## Conflict of Interests

The authors declare no conflict of interest.

## Supporting information

As a service to our authors and readers, this journal provides supporting information supplied by the authors. Such materials are peer reviewed and may be re‐organized for online delivery, but are not copy‐edited or typeset. Technical support issues arising from supporting information (other than missing files) should be addressed to the authors.

Supporting Information

## Data Availability

The data that support the findings of this study are available from the corresponding author upon reasonable request.
