# Supplementary material for: Optimizing DSSCs Performance for Indoor Lighting: Matching Organic Dyes Absorption and Indoor Lamps Emission Profiles to Maximize Efficiency
Source: ChemistryOpen. 2025 Jan 28;14(7):e202400464. doi: 10.1002/open.202400464 (PMC13140842; doi:10.1002/open.202400464)
Supplement: Supplementary file 1 — Supporting Information [file OPEN-14-e202400464-s001.pdf]

ChemistryOpen

Supporting Information

Optimizing DSSCs Performance for Indoor Lighting: Matching Organic Dyes Absorption and Indoor Lamps Emission Profiles to Maximize Efficiency

Giorgia Salerno, Daniele Franchi, Alessio Dessì, Matteo Bartolini, Norberto Manfredi, Alessandro Abbotto,* and Ottavia Bettucci*

Supporting Information

Optimizing DSSCs Performance for Indoor Lighting: Matching Organic Dyes Absorption and Indoor Lamps Emission Profiles to Maximize Efficiency

Giorgia Salerno, Daniele Franchi, Alessio Dessì, Matteo Bartolini, Norberto Manfredi, and Alessandro Abbotto,* Ottavia Bettucci*

Figure S1. Normalized UV-Vis spectra of dyes as TiO_2 films (Solaronix R/SP 1 µm). 3

Figure S2. Sensitized FTO/TiO_2 films of dyes (from left to right: **L1**, **S1**, **Y123**, **TP1**). 3

Table S1. Absorption peaks of the dye-sensitized FTO/TiO_2 films. 3

Figure S3. Emission spectra of the light sources in the wavelength range 350 - 800 nm, measured by using a Hamamatsu C10082CAH spectrophotometer and a power meter (Thorlabs PM100USB power and energy meter) equipped with a photodiode just calibrated for the purpose (Si-photodiode S120VC, recalibrated 03/23 by ReRa Solutions). 4

Figure S4. Superposition between the normalized (emission peak) emission spectra of the lamps (left, OSRAM 930; right, OSRAM 765) and the UV-Vis absorption spectra of the dyes. 4

Table S2. Calculated weighted values of the extinction coefficient ε' for dye **L1**, recalibrated according to the emission spectrum of lamp OSRAM 765 (350 – 800 nm region). 5

Figure S5. Lamp-corrected absorption spectra (weighted molar absorptivity ε' vs λ) of dyes in solution (CH_2Cl_2). 14

Figure S6. Thickness profile of TiO_2 printing for the fabrication of the DSSC photoanode. 15

Synthesis of Cu-based redox couples. 15

Figure S7. Cyclic voltammetry of $Cu^{I}(tmby)_2TFSI$ (red) and $Cu^{II}(tmby)_2(TFSI)_2$ (black) in a 0.1 M tetrabutylammonium perchlorate solution in CH_2Cl_2 as a supporting electrolyte using a glassy carbon working electrode, a Pt wire as a counter electrode, and an Ag/$AgNO_3$ in 0.1 M tetrabutylammonium perchlorate solution in CH_3CN as a pseudo-reference electrode. 16

Figure S8. UV-Vis spectra of the Cu^{I}/Cu^{II} complexes in CH_3CN solution. 16

Table S3. Absorption peaks of Cu^{I}/ Cu^{II} complexes in CH_3CN. 16

Figure S9. *J/V* curves of DSSCs (1 sun, AM 1.5G). 17

Table S4. Photovoltaic characteristics of sensitized DSSC under AM 1.5G sun-simulated light.[a].. 17

Figure S10. Homemade set-up of the fluorescent tube OSRAM 930 used in this work. 18

Figure S11. Reflectance measurement relative to the material with which the low-light measurement set-up was coated (solid green line) in comparison with the emission spectra of the lamps (solid black line). 18

Evaluation of synthetic accessibility and cost. 19

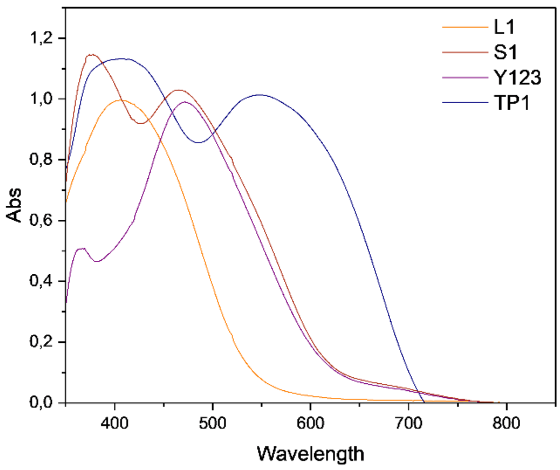

Figure S1. Normalized UV-Vis spectra of dyes as TiO_2 films (Solaronix R/SP 1 µm).

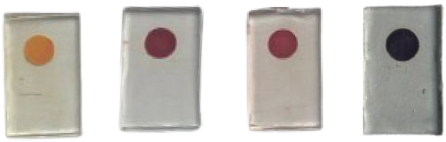

Figure S2. Sensitized FTO/TiO_2 films of dyes (from left to right: **L1**, **S1**, **Y123**, **TP1**).

Table S1. Absorption peaks of the dye-sensitized FTO/TiO_2 films.

**Dye**	**λ_{max} (nm)**
**L1**	403
**S1**	464
**Y123**	471
**TP1**	546

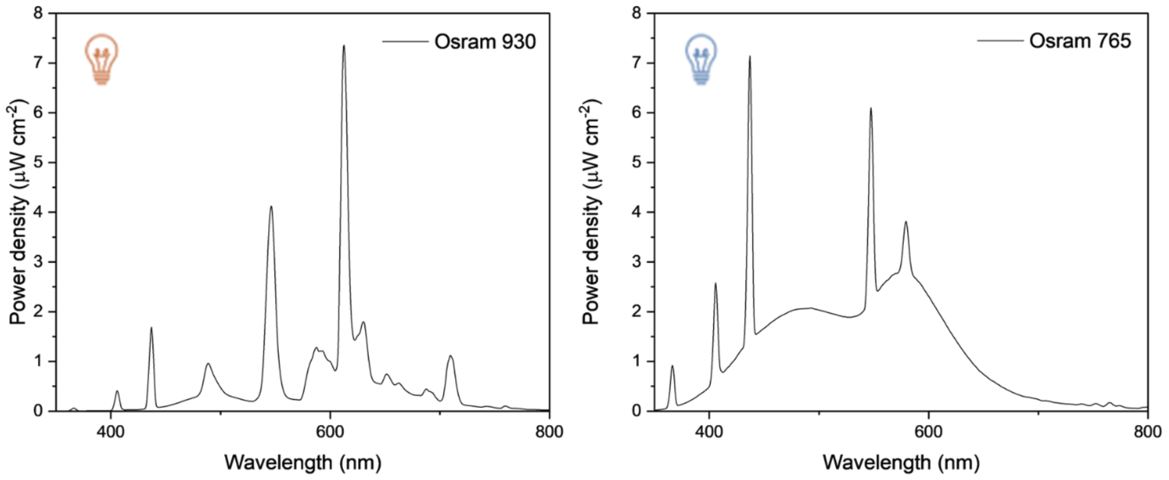

Figure S3. Emission spectra of the light sources in the wavelength range 350 - 800 nm, measured by using a Hamamatsu C10082CAH spectrophotometer and a power meter (Thorlabs PM100USB power and energy meter) equipped with a photodiode just calibrated for the purpose (Si-photodiode S120VC, recalibrated 03/23 by ReRa Solutions).

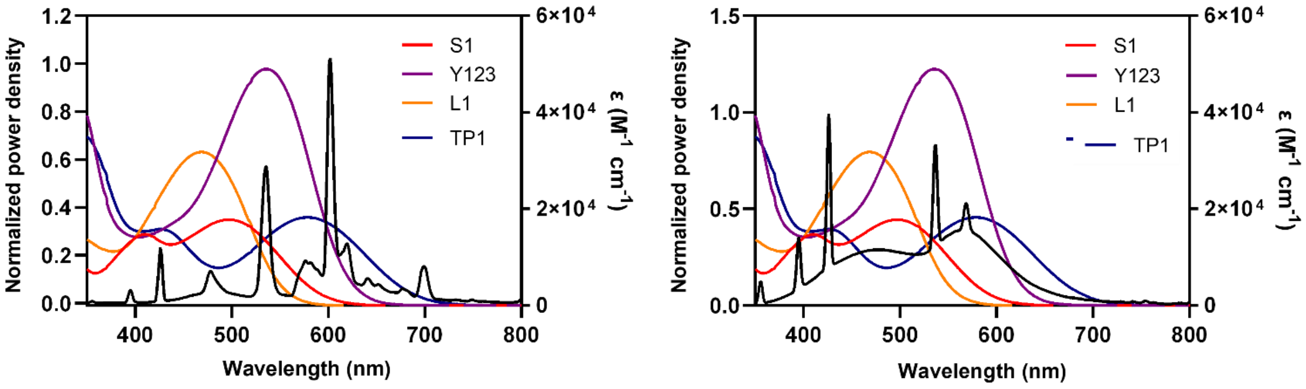

Figure S4. Superposition between the normalized (emission peak) emission spectra of the lamps (left, OSRAM 930; right, OSRAM 765) and the UV-Vis absorption spectra of the dyes.

Table S2. Calculated weighted values of the extinction coefficient ε' for dye **L1**, recalibrated according to the emission spectrum of lamp OSRAM 765 (350 – 800 nm region).

λ (nm)	ε (M^{-1} cm^{-1})[a]	lamp normalized emission intensity[b]	ε' (M^{-1} cm^{-1})[c]
800	0	0.01	0
799	0	0.01	0
798	0	0.01	0
797	0	0.01	0
796	0	0.01	0
795	0	0.01	0
794	0	0.01	0
793	0	0.01	0
792	0	0.01	0
791	0	0.01	0
790	0	0.01	0
789	0	0.01	0
788	0	0.01	0
787	0	0.01	0
786	0	0.01	0
785	0	0.01	0
784	0	0.01	0
783	0	0.01	0
782	0	0.01	0
781	0	0.01	0
780	0	0.01	0
779	0	0.01	0
778	0	0.01	0
777	0	0.01	0
776	0	0.01	0
775	0	0.01	0
774	0	0.02	0
773	0	0.02	0
772	0	0.01	0
771	0	0.01	0
770	0	0.01	0
769	0	0.02	0
768	0	0.02	0
767	0	0.02	0
766	0	0.02	0
765	0	0.02	0
764	0	0.02	0
763	0	0.02	0
762	0	0.02	0
761	0	0.02	0
760	0	0.01	0
759	0	0.01	0
758	0	0.01	0
757	0	0.02	0
756	0	0.02	0
755	0	0.02	0
754	0	0.02	0

753	0	0.02	0
752	0	0.02	0
751	0	0.02	0
750	0	0.02	0
749	0	0.02	0
748	0	0.02	0
747	0	0.02	0
746	0	0.02	0
745	0	0.02	0
744	0	0.02	0
743	0	0.02	0
742	0	0.02	0
741	0	0.02	0
740	0	0.02	0
739	0	0.02	0
738	0	0.02	0
737	0	0.02	0
736	0	0.02	0
735	0	0.02	0
734	0	0.02	0
733	0	0.02	0
732	0	0.02	0
731	0	0.02	0
730	0	0.02	0
729	0	0.02	0
728	0	0.02	0
727	0	0.02	0
726	0	0.02	0
725	0	0.02	0
724	0	0.02	0
723	0	0.02	0
722	0	0.02	0
721	0	0.02	0
720	0	0.02	0
719	0	0.02	0
718	0	0.02	0
717	0	0.02	0
716	0	0.02	0
715	0	0.02	0
714	0	0.02	0
713	0	0.03	0
712	0	0.03	0
711	0	0.03	0
710	0	0.03	0
709	0	0.03	0
708	0	0.03	0
707	0	0.03	0
706	0	0.03	0
705	0	0.03	0
704	0	0.03	0
703	0	0.03	0
702	0	0.03	0
701	0	0.03	0
700	0	0.03	0

699	0	0.03	0
698	0	0.03	0
697	0	0.04	0
696	0	0.04	0
695	0	0.04	0
694	0	0.04	0
693	0	0.04	0
692	0	0.04	0
691	0	0.04	0
690	0	0.04	0
689	0	0.04	0
688	0	0.04	0
687	0	0.04	0
686	0	0.04	0
685	0	0.04	0
684	0	0.04	0
683	0	0.05	0
682	0	0.05	0
681	0	0.05	0
680	0	0.05	0
679	0	0.05	0
678	0	0.05	0
677	0	0.05	0
676	0	0.05	0
675	0	0.06	0
674	0	0.06	0
673	0	0.06	0
672	0	0.06	0
671	0	0.06	0
670	0	0.06	0
669	0	0.07	0
668	0	0.07	0
667	0	0.07	0
666	0	0.07	0
665	0	0.07	0
664	0	0.07	0
663	0	0.08	0
662	0	0.08	0
661	0	0.08	0
660	0	0.08	0
659	0	0.08	0
658	0	0.09	0
657	0	0.09	0
656	0	0.09	0
655	0	0.09	0
654	0	0.10	0
653	0	0.10	0
652	0	0.10	0
651	0	0.10	0
650	0	0.11	0
649	0	0.11	0
648	0	0.11	0
647	0	0.11	0
646	0	0.12	0

645	0	0.12	0
644	0	0.12	0
643	0	0.13	0
642	0	0.13	0
641	0	0.13	0
640	0	0.14	0
639	0	0.14	0
638	0	0.15	0
637	0	0.15	0
636	0	0.15	0
635	0	0.16	0
634	0	0.16	0
633	0	0.17	0
632	0	0.17	0
631	0	0.17	0
630	0	0.18	0
629	0	0.18	0
628	0	0.19	0
627	0	0.19	0
626	0	0.20	0
625	0	0.20	0
624	0	0.21	0
623	0	0.21	0
622	0	0.21	0
621	0	0.22	0
620	0	0.23	0
619	0	0.23	0
618	0	0.24	0
617	0	0.24	0
616	0	0.25	0
615	0	0.25	0
614	5	0.26	1
613	9	0.26	2
612	15	0.27	4
611	21	0.27	6
610	26	0.28	7
609	32	0.28	9
608	38	0.29	11
607	44	0.29	13
606	51	0.30	15
605	60	0.30	18
604	68	0.31	21
603	76	0.31	24
602	86	0.32	27
601	96	0.32	31
600	107	0.33	35
599	118	0.33	39
598	130	0.34	44
597	143	0.34	49
596	159	0.35	55
595	176	0.35	62
594	194	0.36	69
593	215	0.36	78
592	235	0.37	86

591	258	0.37	95
590	282	0.37	105
589	307	0.38	116
588	336	0.38	127
587	366	0.38	140
586	397	0.39	154
585	431	0.40	170
584	466	0.41	192
583	504	0.43	219
582	545	0.47	258
581	589	0.51	301
580	634	0.53	339
579	686	0.54	370
578	740	0.52	385
577	797	0.49	394
576	857	0.45	390
575	922	0.43	392
574	991	0.41	405
573	1065	0.40	424
572	1144	0.39	451
571	1226	0.39	482
570	1313	0.39	516
569	1408	0.39	552
568	1508	0.39	588
567	1615	0.39	627
566	1727	0.39	665
565	1847	0.38	705
564	1973	0.38	746
563	2107	0.37	789
562	2247	0.37	838
561	2395	0.37	887
560	2551	0.37	937
559	2715	0.36	988
558	2886	0.36	1039
557	3063	0.36	1090
556	3251	0.35	1144
555	3447	0.35	1197
554	3653	0.34	1254
553	3871	0.34	1327
552	4099	0.36	1476
551	4338	0.43	1859
550	4589	0.57	2603
549	4852	0.74	3572
548	5123	0.84	4283
547	5408	0.85	4586
546	5704	0.74	4209
545	6015	0.57	3445
544	6333	0.44	2763
543	6666	0.36	2376
542	7010	0.30	2115
541	7364	0.29	2117
540	7732	0.28	2198
539	8110	0.28	2279
538	8498	0.28	2361

537	8898	0.28	2452
536	9308	0.27	2548
535	9730	0.27	2651
534	10164	0.27	2759
533	10608	0.27	2869
532	11062	0.27	2986
531	11525	0.27	3101
530	11995	0.27	3223
529	12476	0.27	3344
528	12963	0.27	3475
527	13456	0.27	3608
526	13955	0.27	3744
525	14462	0.27	3884
524	14974	0.27	4031
523	15491	0.27	4173
522	16008	0.27	4320
521	16533	0.27	4474
520	17743	0.27	4817
519	18077	0.27	4921
518	18406	0.27	5022
517	18726	0.27	5133
516	19211	0.27	5278
515	19737	0.28	5440
514	20255	0.28	5603
513	20772	0.28	5766
512	21287	0.28	5922
511	21792	0.28	6080
510	22293	0.28	6234
509	22789	0.28	6397
508	23277	0.28	6552
507	23755	0.28	6711
506	24225	0.28	6864
505	24689	0.28	7022
504	25141	0.29	7174
503	25581	0.29	7319
502	26011	0.29	7462
501	26436	0.29	7610
500	26851	0.29	7742
499	27251	0.29	7882
498	27640	0.29	8019
497	28019	0.29	8145
496	28388	0.29	8274
495	28745	0.29	8408
494	29084	0.29	8533
493	29413	0.29	8650
492	29728	0.29	8738
491	30033	0.29	8815
490	30322	0.29	8886
489	30604	0.29	8958
488	30865	0.29	9031
487	31119	0.29	9111
486	31360	0.29	9170
485	31588	0.29	9232
484	31802	0.29	9294

483	32000	0.29	9342
482	32185	0.29	9384
481	32359	0.29	9419
480	32517	0.29	9450
479	32664	0.29	9472
478	32796	0.29	9497
477	32919	0.29	9497
476	33032	0.29	9497
475	33126	0.29	9486
474	33204	0.29	9477
473	33271	0.28	9447
472	33325	0.28	9407
471	33363	0.28	9361
470	33388	0.28	9317
469	33397	0.28	9262
468	33395	0.28	9206
467	33375	0.27	9160
466	33351	0.27	9103
465	33307	0.27	9034
464	33255	0.27	8966
463	33195	0.27	8875
462	33117	0.27	8790
461	33030	0.26	8697
460	32925	0.26	8603
459	32806	0.26	8498
458	32676	0.26	8387
457	32532	0.25	8279
456	32382	0.25	8160
455	32223	0.25	8040
454	32051	0.25	7907
453	31875	0.24	7772
452	31689	0.24	7645
451	31488	0.24	7528
450	31271	0.24	7411
449	31053	0.23	7277
448	30823	0.23	7150
447	30576	0.23	7012
446	30317	0.23	6875
445	30057	0.22	6735
444	29790	0.22	6599
443	29509	0.22	6476
442	29225	0.22	6418
441	28930	0.24	7084
440	28639	0.37	10559
439	28338	0.63	17805
438	28027	0.88	24729
437	27710	1.00	27710
436	27391	0.91	24939
435	27070	0.66	17969
434	26740	0.44	11748
433	26407	0.29	7626
432	26068	0.21	5374
431	25728	0.18	4733
430	25385	0.18	4511

429	25033	0.17	4356
428	24681	0.17	4198
427	24321	0.17	4021
426	23962	0.16	3847
425	23598	0.16	3678
424	23234	0.15	3504
423	22863	0.15	3342
422	22492	0.14	3204
421	22121	0.14	3077
420	21297	0.14	2876
419	21062	0.13	2758
418	20819	0.13	2667
417	20567	0.13	2573
416	20224	0.12	2466
415	19857	0.12	2348
414	19495	0.11	2238
413	19127	0.11	2137
412	18771	0.11	2078
411	18420	0.11	2093
410	18071	0.12	2166
409	17719	0.15	2731
408	17381	0.23	4020
407	17048	0.31	5345
406	16719	0.36	6058
405	16394	0.33	5491
404	16079	0.25	4031
403	15774	0.17	2659
402	15477	0.11	1727
401	15187	0.08	1248
400	14906	0.07	1037
399	14638	0.07	975
398	14379	0.06	913
397	14129	0.06	857
396	13891	0.06	805
395	13662	0.06	760
394	13450	0.05	717
393	13249	0.05	679
392	13064	0.05	641
391	12887	0.05	604
390	12726	0.04	567
389	12574	0.04	531
388	12443	0.04	498
387	12319	0.04	467
386	12208	0.04	439
385	12114	0.03	413
384	12033	0.03	389
383	11965	0.03	365
382	11911	0.03	344
381	11865	0.03	323
380	11830	0.03	303
379	11803	0.02	285
378	11791	0.02	269
377	11788	0.02	254
376	11792	0.02	240

375	11804	0.02	227
374	11829	0.02	216
373	11857	0.02	205
372	11892	0.02	196
371	11937	0.02	197
370	12054	0.02	285
369	12099	0.05	580
368	12151	0.09	1078
367	12204	0.12	1480
366	12282	0.13	1566
365	12372	0.10	1294
364	12465	0.07	812
363	12572	0.03	432
362	12687	0.02	219
361	12813	0.01	119
360	12946	0.01	88
359	13082	0.01	79
358	13224	0.01	72
357	13383	0.00	67
356	13529	0.00	62
355	13675	0.00	56
354	13823	0.00	52
353	13960	0.00	46
352	14087	0.00	41
351	14201	0.00	38
350	14305	0.00	35

[a] Real extinction coefficient values (ε) at each wavelength. [b] The emission spectrum of the lamp is scaled so that its intensity at its emission peak (437 nm) is normalized to 1. [c] Weighted extinction coefficient values (ε’) calculated at each wavelength by multiplying the real ε value at that wavelength by the normalized emission intensity of the lamp at the same wavelength (from the previous two columns). Since the normalized intensity at the lamp's emission peak (437 nm) is equal to 1, ε = ε’ at this wavelength.

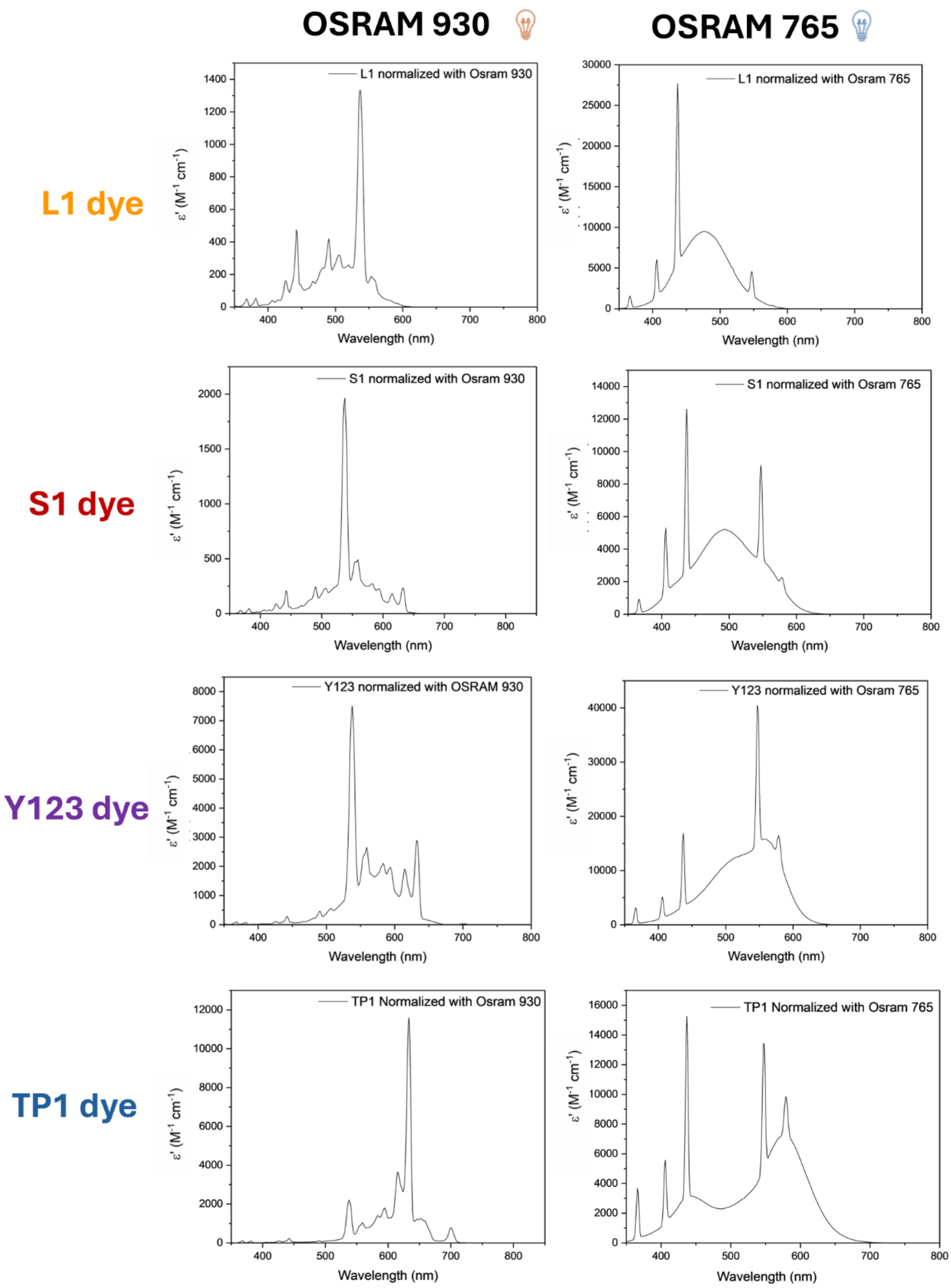

Figure S5. Lamp-corrected absorption spectra (weighted molar absorptivity ε' vs λ) of dyes in solution (CH_2Cl_2).

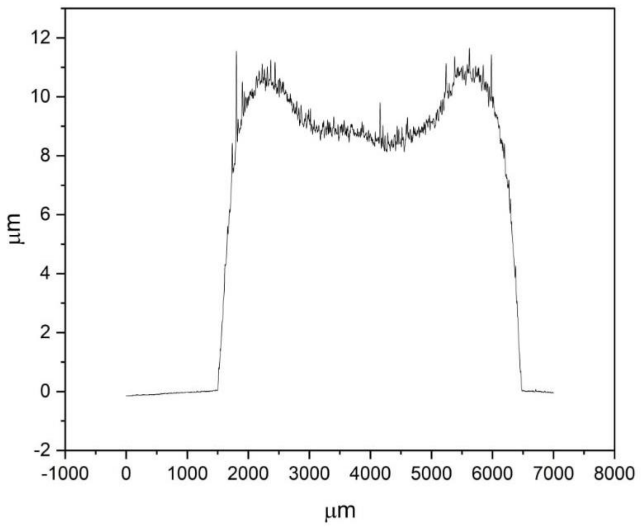

Figure S6. Thickness profile of TiO_2 printing for the fabrication of the DSSC photoanode.

Synthesis of Cu-based redox couples.

$Cu^{I}(tmby)_2TFSI$. One equivalent of CuI (140 mg, 0.73 mmol) was mixed with 2 equivalents of 4,4',6,6'-tetramethyl-2,2'-bipyridine (tmby) (312 mg, 1.47 mmol) in 30 mL dry ethanol, under nitrogen atmosphere, at room temperature for 2 hours. The complex was obtained as an intense red crystalline powder. The product was filtered and redissolved by adding of 5 mL of deionized water followed by an addition of 2 equivalents of lithium bis(trifluoromethanesulfonyl)imide (LiTFSI) (422 mg, 1.47 mmol). The solution was further stirred for 2 h at room temperature and under a nitrogen atmosphere resulting in a red precipitate. The complex was collected by filtration and washed with water. The product was collected as a red powder (143 mg, 25%). λ(max): 450 nm.[19,20]

$Cu^{II}(tmby)_2(TFSI)_2$. One equivalent of $CuCl_2$ (70 mg, 0.521 mmol) was mixed with 2 equivalents of 4,4',6,6'-tetramethyl-2,2'-bipyridine (221 mg, 1.04 mmol) in 30 mL of dry ethanol, under a nitrogen atmosphere, at room temperature for 2 h. The resulted complex was obtained as a yellow solid; the product was washed and dissolved in 5 mL of deionized water with 4 equivalents of LiTFSI (598 mg, 2.08 mmol). The solution was further stirred for 2 h at room temperature and under a nitrogen atmosphere resulting in a violet precipitation. The complex was collected by filtration and washed with water. The product was obtained as a violet powder (89 mg, 16%). λ(max): 732 nm.[19;20]

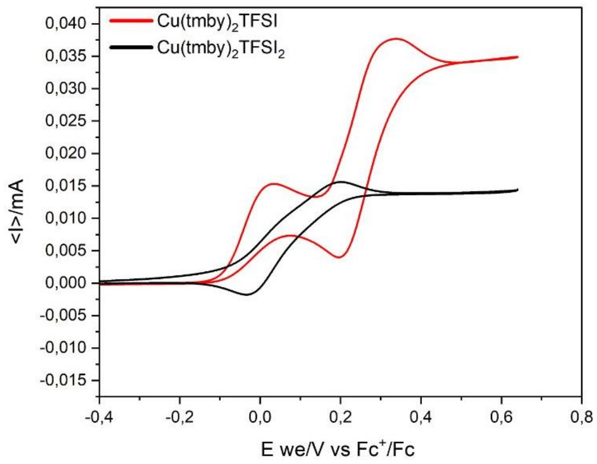

Figure S7. Cyclic voltammetry of $Cu^{I}(tmby)_2TFSI$ (red) and $Cu^{II}(tmby)_2(TFSI)_2$ (black) in a 0.1 M tetrabutylammonium perchlorate solution in CH_2Cl_2 as a supporting electrolyte using a glassy carbon working electrode, a Pt wire as a counter electrode, and an $Ag/AgNO_3$ in 0.1 M tetrabutylammonium perchlorate solution in CH_3CN as a pseudo-reference electrode.

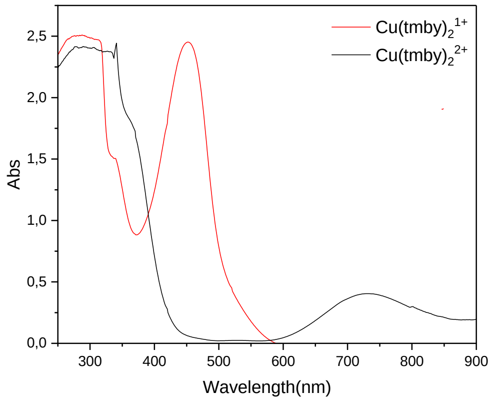

Figure S8. UV-Vis spectra of the Cu^{I}/Cu^{II} complexes in CH_3CN solution.

Table S3. Absorption peaks of Cu^{I}/ Cu^{II} complexes in CH_3CN.

complex	λ_{max} (nm)
$Cu^{I}(tmby)_2TFSI$	451
$Cu^{II}(tmby)_2(TFSI)_2$	729

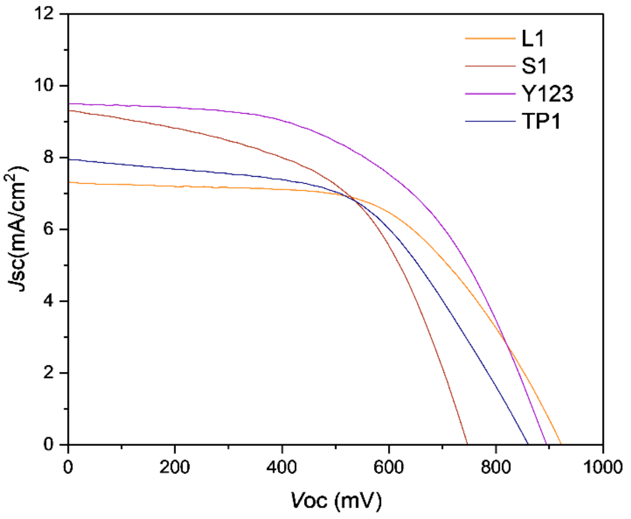

Figure S9. *J/V* curves of DSSCs (1 sun, AM 1.5G).

Table S4. Photovoltaic characteristics of sensitized DSSC under AM 1.5G sun-simulated light.[a]

Dye	V_{oc} (mV)	J_{sc} (mA/cm^2)	FF	PCE (%)
**L1**	944<br>(931 ± 5)	7.2<br>(7.2 ± 0.1)	0.59<br>(0.59 ± 0.02)	4.0<br>(3.9 ± 0.1)
**S1**	814<br>(786 ± 35)	7.8<br>(8.4 ± 0.8)	0.59<br>(0.56 ± 0.37)	3.8<br>(3.8 ± 0.1)
**Y123**	894<br>(897 ± 15)	9.5<br>(9.2 ± 0.3)	0.54<br>(0.54 ± 0.00)	4.6<br>(4.4 ± 0.2)
**TP1**	866<br>(866 ± 5)	7.5<br>(7.4 ± 0.4)	0.56<br>(0.56 ± 0.02)	3.6<br>(3.6 ± 0.1)

[a] Average values over 3 devices in parentheses.

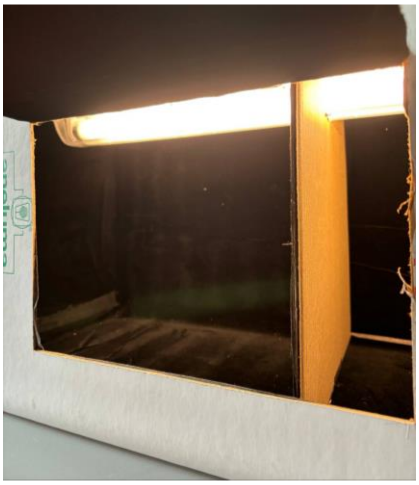

Figure S10. Homemade set-up of the fluorescent tube OSRAM 930 used in this work.

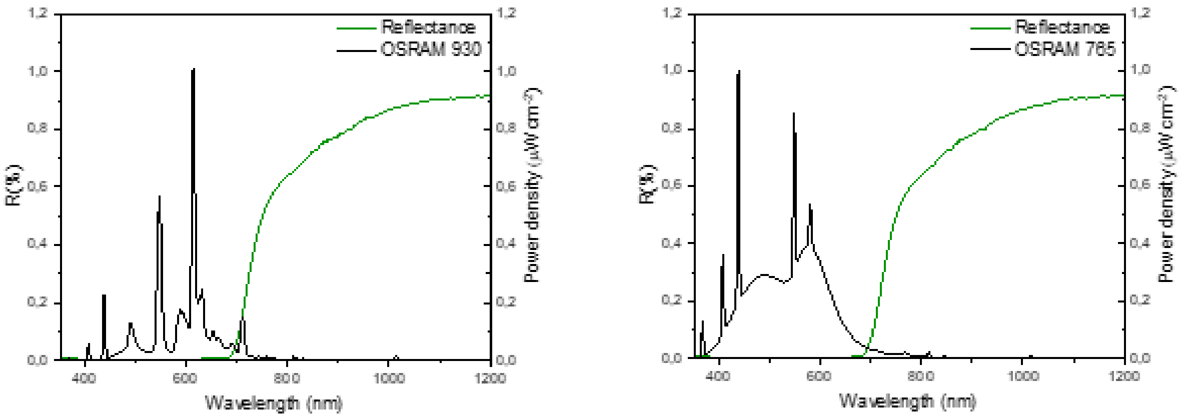

Figure S11. Reflectance measurement relative to the material with which the low-light measurement set-up was coated (solid green line) in comparison with the emission spectra of the lamps (solid black line).

Evaluation of synthetic accessibility and cost.

L1

Literature reference synthesis: Ref. 25a. The reported amounts of reagents of products have been normalized to those of the first step of the reported synthesis.

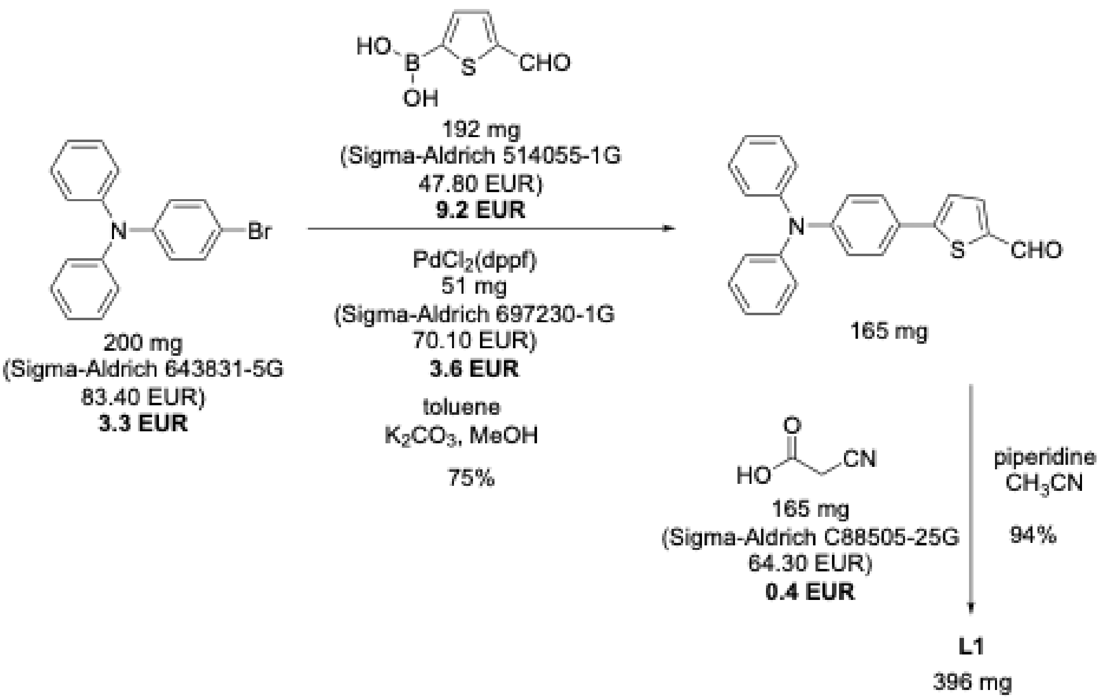

Number of synthetic steps= 2

Total cost for 396 mg = (3.3 + 9.2 + 3.6 + 0.4) EUR = 16.5 EUR

<u>Total cost for 1 g = 41.7 EUR</u>

<u>Total cost for 1 mmol (M_w = 422.50 g/mol) = 17.6 EUR</u>

S1

Literature reference synthesis: Ref. 25b. The reported amounts of reagents of products have been normalized to those of the first step of the reported synthesis.

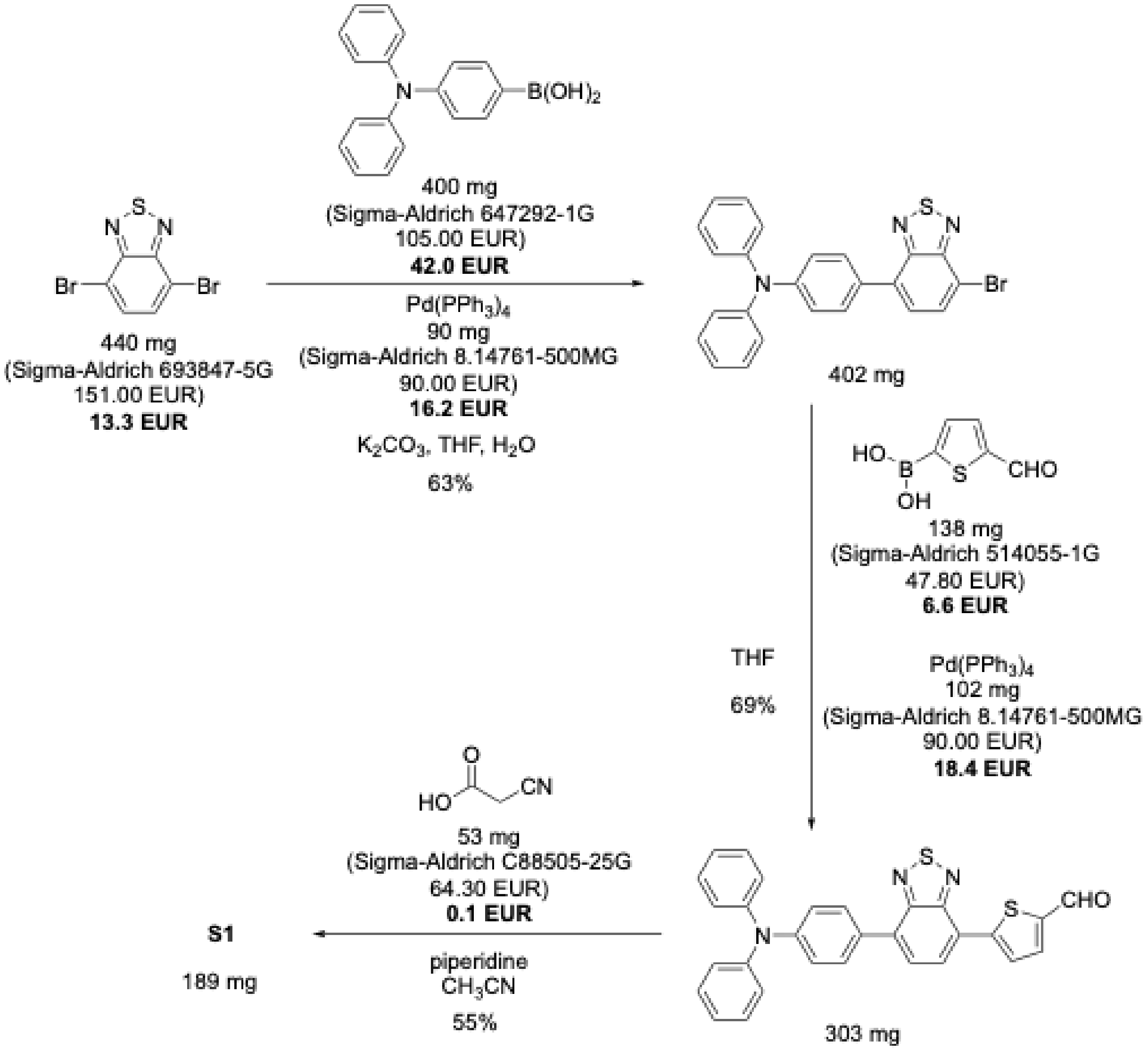

Number of synthetic steps = 3

Total cost for 189 mg = (13.3 + 42.0 + 16.2 + 6.6 + 18.4 + 0.1) EUR = 96.6 EUR

Total cost for 1 g = 511.1 EUR

Total cost for 1 mmol (M_w = 556.66 g/mol) = 284.5 EUR

Y123

Literature reference synthesis: Ref. 25c. The reported amounts of reagents of products have been normalized to those of the first step of the reported synthesis.

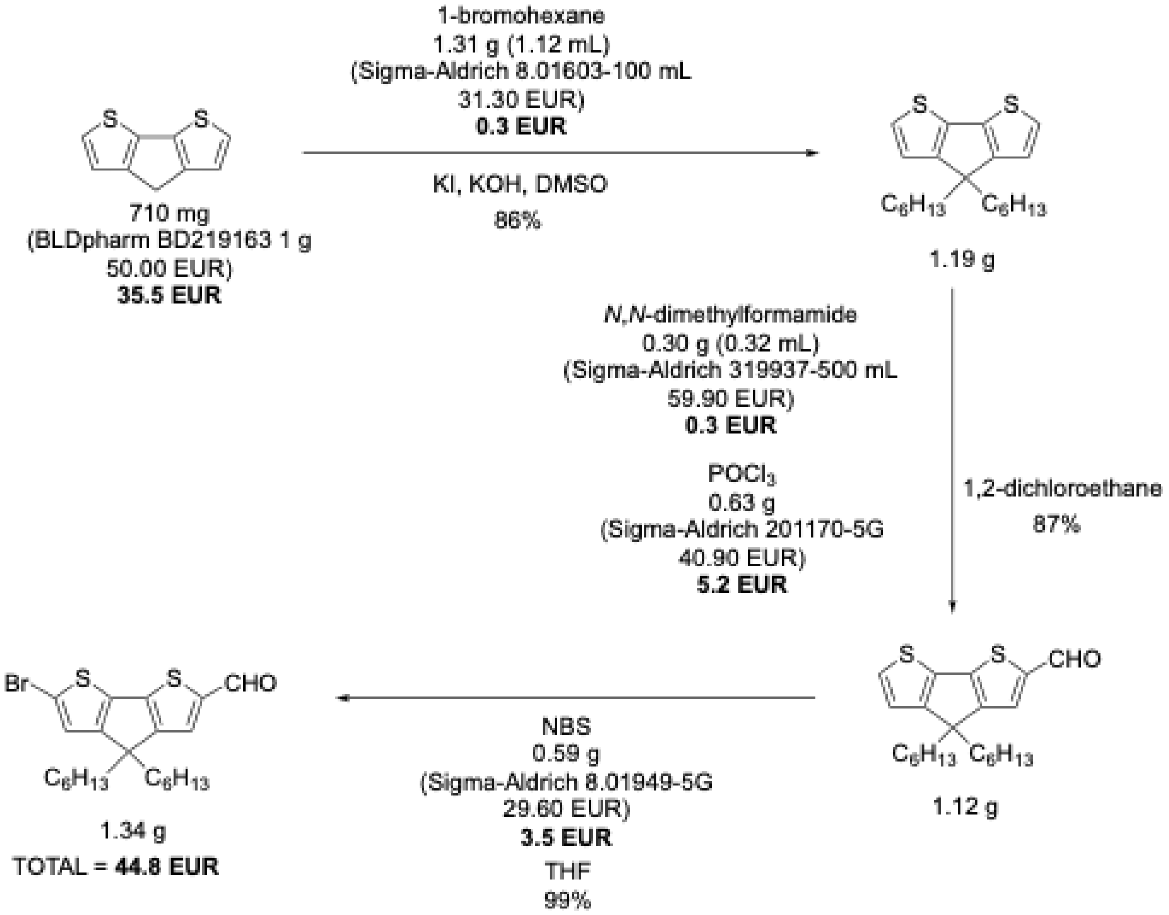

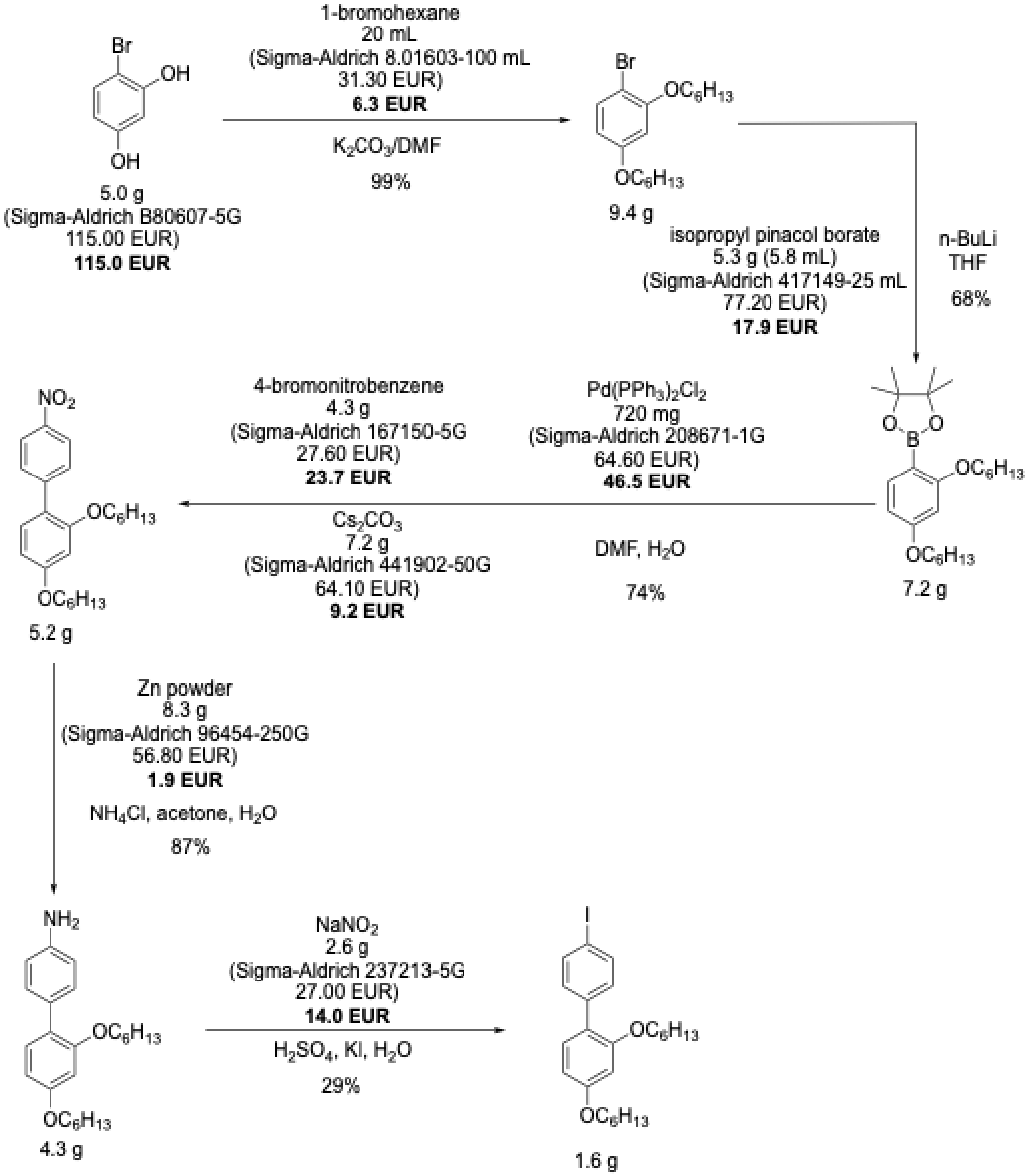
Br
OH
OH
5.0 g
(Sigma-Aldrich B80607-5G
115.00 EUR)
115.0 EUR
1-bromohexane
20 mL
(Sigma-Aldrich 8.01603-100 mL
31.30 EUR)
6.3 EUR
K2CO3/DMF
99%
Br
OC6H13
OC6H13
9.4 g
isopropyl pinacol borate
5.3 g (5.8 mL)
(Sigma-Aldrich 417149-25 mL
77.20 EUR)
17.9 EUR
n-BuLi
THF
68%
O
B
O
OC6H13
OC6H13
7.2 g
Pd(PPh3)2Cl2
720 mg
(Sigma-Aldrich 208671-1G
64.60 EUR)
46.5 EUR
DMF, H2O
74%
4-bromonitrobenzene
4.3 g
(Sigma-Aldrich 167150-5G
27.60 EUR)
23.7 EUR
Cs2CO3
7.2 g
(Sigma-Aldrich 441902-50G
64.10 EUR)
9.2 EUR
NO2
OC6H13
OC6H13
5.2 g
Zn powder
8.3 g
(Sigma-Aldrich 96454-250G
56.80 EUR)
1.9 EUR
NH4Cl, acetone, H2O
87%
NH2
OC6H13
OC6H13
4.3 g
NaNO2
2.6 g
(Sigma-Aldrich 237213-5G
27.00 EUR)
14.0 EUR
H2SO4, KI, H2O
29%
I
OC6H13
OC6H13
1.6 g

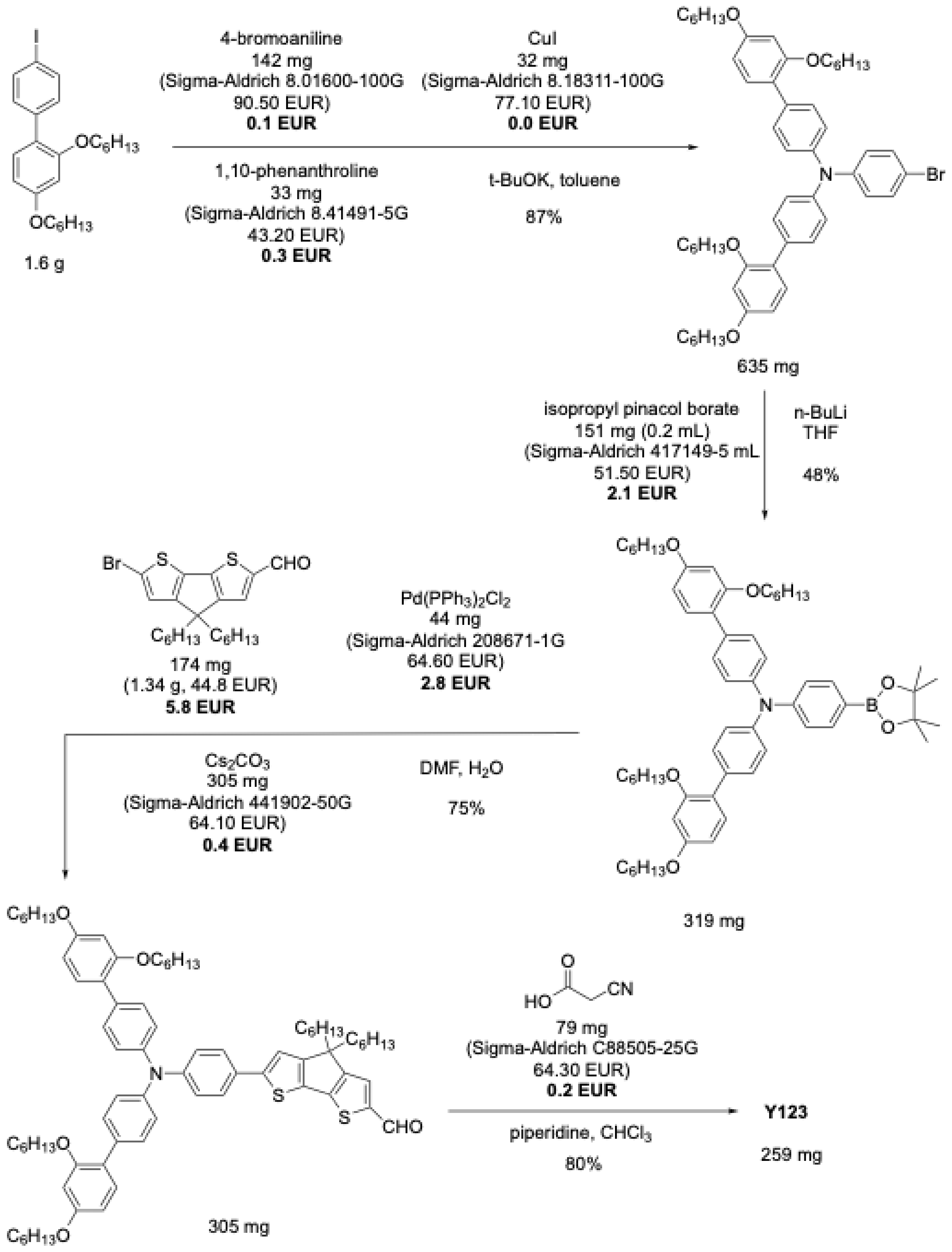

Number of synthetic steps: 12

Total cost for 259 mg = (115.0 + 6.3 + 17.9 + 23.7 + 46.5 + 9.2 + 1.9 + 14.0 + 0.1 + 0.0 + 0.3 + 2.1 +5.8 + 2.8 + 0.4 + 0.2) EUR = 246.2 EUR

Total cost for 1 g = 950.6 EUR

Total cost for 1 mmol (M_w = 1237.80 g/mol) = 1176.6 EUR

TP1

Literature reference synthesis: Ref. 18. The reported amounts of reagents of products have been normalized to those of the first step of the reported synthesis.

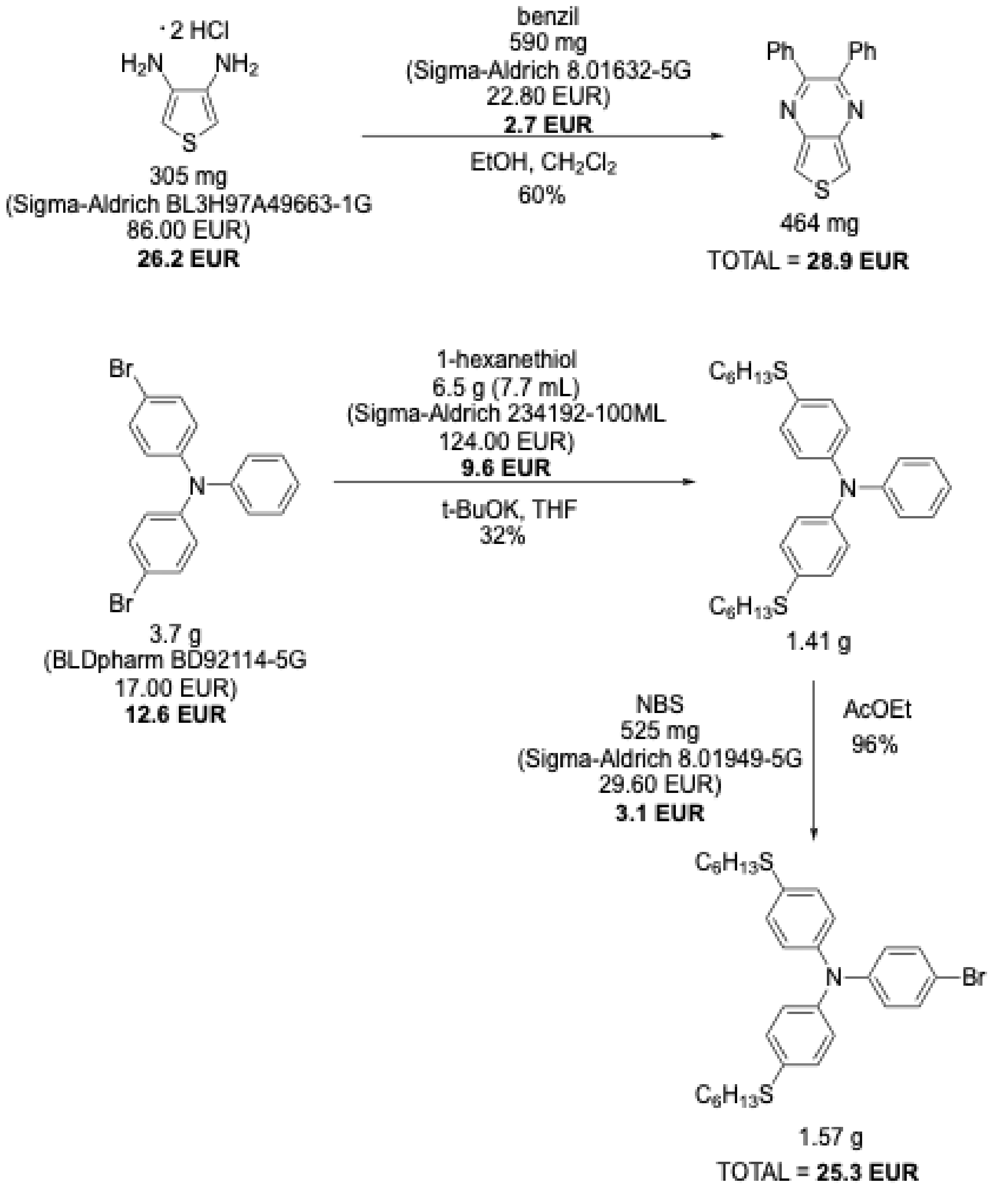

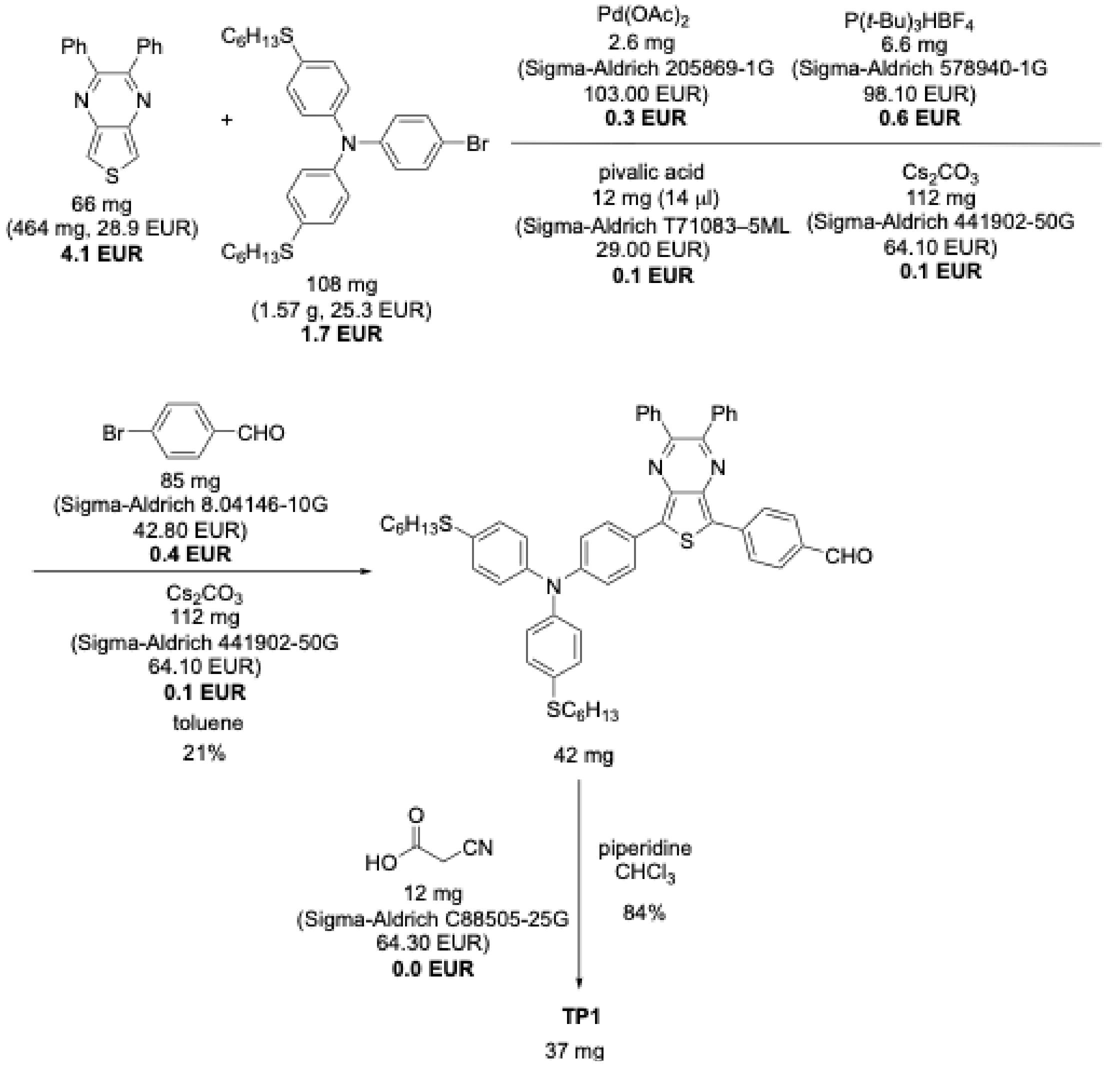

Number of synthetic steps: 6

Total cost for 37 mg = (4.1 + 1.7 + 0.3 + 0.6 + 0.1 + 0.1 + 0.4 + 0.1 + 0.0) EUR = 7.4 EUR

Total cost for 1 g = 200.0 EUR

Total cost for 1 mmol (M_w = 935.28 g/mol) = 187.1 EUR
